# The hypothalamic steroidogenic pathway mediates susceptibility to inflammation-evoked depression in female mice

**DOI:** 10.1186/s12974-023-02976-7

**Published:** 2023-12-07

**Authors:** Fu-Rong Xu, Zhen-Hong Wei, Xiao-Xia Xu, Xiao-Gang Zhang, Chao-Jun Wei, Xiao-Ming Qi, Yong-Hong Li, Xiao-Ling Gao, Yu Wu

**Affiliations:** 1 NHC Key Laboratory of Diagnosis and Therapy of Gastrointestinal Tumor, Gansu Provincial Hospital, Lanzhou, 730000 China; 2 The Clinical Laboratory Center, Hainan General Hospital, Hainan Affiliated Hospital of Hainan Medical University, Haikou, 570100 China; 3 Department of Nursing, The Second People’s Hospital of Wuwei, Wuwei, 733000 China; 4 Department of Nursing, People’s Hospital of Wuwei, Wuwei, 733000 China; 5 Institute of Clinical Research and Translational Medicine, Gansu Provincial Hospital, Lanzhou, 730000 China; 6 School of Basic Medical Sciences, Southern Medical University, Guangzhou, 510515 China; 7 School of Psychology, Shenzhen University, Shenzhen, 518060 China

**Keywords:** Depression, Sex, Neuroinflammation, Hypothalamus, Neurosteroid biosynthesis, Pregnenolone

## Abstract

**Background:**

Depression is two-to-three times more frequent among women. The hypothalamus, a sexually dimorphic area, has been implicated in the pathophysiology of depression. Neuroinflammation-induced hypothalamic dysfunction underlies behaviors associated with depression. The lipopolysaccharide (LPS)-induced mouse model of depression has been well-validated in numerous laboratories, including our own, and is widely used to investigate the relationship between neuroinflammation and depression. However, the sex-specific differences in metabolic alterations underlying depression-associated hypothalamic neuroinflammation remain unknown.

**Methods:**

Here, we employed the LPS-induced mouse model of depression to investigate hypothalamic metabolic changes in both male and female mice using a metabolomics approach. Through bioinformatics analysis, we confirmed the molecular pathways and biological processes associated with the identified metabolites. Furthermore, we employed quantitative real-time PCR, enzyme-linked immunosorbent assay, western blotting, and pharmacological interventions to further elucidate the underlying mechanisms.

**Results:**

A total of 124 and 61 differential metabolites (DMs) were detected in male and female mice with depressive-like behavior, respectively, compared to their respective sex-matched control groups. Moreover, a comparison between female and male model mice identified 37 DMs. We capitalized on biochemical clustering and functional enrichment analyses to define the major metabolic changes in these DMs. More than 55% of the DMs clustered into lipids and lipid-like molecules, and an imbalance in lipids metabolism was presented in the hypothalamus. Furthermore, steroidogenic pathway was confirmed as a potential sex-specific pathway in the hypothalamus of female mice with depression. Pregnenolone, an upstream component of the steroid hormone biosynthesis pathway, was downregulated in female mice with depressive-like phenotypes but not in males and had considerable relevance to depressive-like behaviors in females. Moreover, exogenous pregnenolone infusion reversed depressive-like behaviors in female mice with depression. The 5α-reductase type I (SRD5A1), a steroidogenic hub enzyme involved in pregnenolone metabolism, was increased in the hypothalamus of female mice with depression. Its inhibition increased hypothalamic pregnenolone levels and ameliorated depressive-like behaviors in female mice with depression.

**Conclusions:**

Our study findings demonstrate a marked sexual dimorphism at the metabolic level in depression, particularly in hypothalamic steroidogenic metabolism, identifying a potential sex-specific pathway in female mice with depressive-like behaviors.

**Supplementary Information:**

The online version contains supplementary material available at 10.1186/s12974-023-02976-7.

## Background

Depression, a debilitating mental illness, is characterized by persistent negative mood, diminished interests and altered cognitive functions [1]. Preclinical and clinical research has implicated immune dysregulation in depression [2, 3]. Systemic inflammation contributes to depression development and even suicidality in some individuals [4]. Subjects with chronic inflammatory diseases, such as systemic infections, arthritis and diabetes mellitus, are three to four times more likely to experience major depressive disorder (MDD) [2, 5]. Compelling evidences highlighted that increased production of inflammatory markers, such as C-reactive protein (CRP), interleukin (IL)-1β, IL-6, and tumor necrosis factor-alpha (TNF-α), occurred in the peripheral circulation or central nervous system (CNS) of patients with MDD compared to healthy controls [6, 7]. Cytokines infiltrated into the brain and modulate pathophysiological processes involved in depression, including neuroendocrine function [8], synaptic plasticity [9], lipids metabolism, and neurotransmitter production and release [10, 11]. Although inflammatory cytokines evoke abnormalities in these biological processes that may underlie the development and maintenance of depression, the exact molecular mechanisms remain elusive and urgently need to be elucidated.

Women experience depression 2–3 times more often than men and exhibit longer symptom severity, greater functional impairment, more atypical depressive symptoms, more frequent recurrences, and less efficient responses to antidepressants [12–14]. Sex biases in depression prevalence have been attributed to differences in the anatomy and function of the human brain, the sexually dimorphic hormonal milieu [15, 16], and dissimilarities in the inflammatory stress responsiveness [17]. Previous studies have demonstrated that women appear to be more susceptible and more vulnerable to mood disorders following systemic inflammation [17]. Certain autoimmune disorders, such as rheumatoid arthritis and multiple sclerosis, exhibit a greater susceptibility to co-morbid depression and a higher prevalence among females in comparison to males [5, 18–20]. Specifically, women with rheumatoid arthritis have elevated CRP and IL-6 levels, which contribute to the heightened risk of depression in female patients [19, 21]. In addition, sex differences exist in the hypothalamic–pituitary–adrenal (HPA) axis [22, 23], which is one of the causes of sex-dependent stress responses [24]. Activation of the HPA axis differs in male and female patients with MDD [13, 24, 25]. In addition, it has been suggested that antidepressant treatments targeting HPA axis-related compounds should consider sex [25]. However, sex differences in HPA axis-based dysregulation in inflammation-associated depression remain unclear.

The hypothalamus, a well-reported sexually dimorphic brain area and key neuroendocrine region of the HPA axis, is closely related to the pathogenesis of depression [25]. Many studies on the hypothalamus in preclinical depression models and patients with depression have found changes at the neuronal and molecular levels. To elucidate the hypothalamic mechanisms underlying inflammation-induced depression using animal models, it is imperative to establish the ability of inflammatory cytokines to elicit depression-like behavior in the models animals. Lipopolysaccharide (LPS), a structural element of outer membrane of Gram-negative bacteria, has been confirmed to induce neuroinflammation and elicit depressive-like behaviors [5, 8, 9, 26]. The LPS-induced depressive-like model is used as a representative model for inflammation-associated depression, a commonly observed phenotype in individuals displaying depressive symptoms. The clinical manifestations of inflammation-mediated depression underscore the significance of immune dysregulation or activation of the inflammatory system in the pathophysiology of depression [2, 3, 6]. In response to LPS-induced depressive-like behavior in rats, glucocorticoid receptor-mediated neuroendocrine responses may modulate hypothalamic cellular mechanisms in a sex-dependent manner [8]. Previously, we have identified disruptions in purine metabolism, ephrin receptor signaling, and glutamatergic transmission in the hypothalamus of male mice with inflammation-induced depressive-like behaviors [9, 26]. To date, sex differences in metabolic alterations in the hypothalamus of mice with depressive-like behavior under inflammatory stress have not been evaluated.

In this study, we hypothesized that the hypothalamic metabolite signatures of inflammation-associated depression differ by sex. We employed a well-validated mouse model of inflammation-induced depression [9, 10, 27] and performed ultra-high-performance liquid chromatography with high-resolution tandem mass spectrometry (UHPLC–HRMS/MS) metabolomics to profile the hypothalamus-based metabolic patterns in depressive-like male and female mice. Multivariate statistical analysis and bioinformatics were used to assess sexually distinctive metabolic profiles, hub metabolites, and signaling pathways in mice with depression under inflammatory stress conditions. In addition, pharmacological interventions combined with conventional differential gene and protein expression analysis approaches offer data validation for signaling pathways and are expected to identify specific hypothalamus-based metabolites and pathways associated with sex differences in depression. Our findings may contribute to the future development of a sex-specific diagnostic test for depression and provide insights into the mechanisms associated with a depression pathogenesis that preferentially affects the female sex.

## Materials and methods

### Animals

Adult male and female C57BL/6 J mice (approximately 10 weeks of age with a body weight range of 22–30 g) were obtained from the Beijing Vital River Laboratory animal technology Co., Ltd. (Beijing, China). All mice were housed under controlled illumination and acclimated for one week under the following environmental conditions: housed in standard shoebox cages, food and water ad libitum, a light–dark cycle of 12 h (lights on at 08:00 a.m. and off at 08:00 p.m.), 50 ± 5% relative humidity, and 24 ± 1 °C room temperature. To minimize circadian rhythm changes and subjective influences on behavioral tests, the mice in each group were intermixed during the observation period, and the behavioral observers were blind to the intervention conditions or group assignment.

A fresh solution of lipopolysaccharide (LPS; L-3129, *Escherichia coli* serotype 0127:B8, Sigma-Aldrich, USA) was prepared in sterile endotoxin-free isotonic saline. The experimental groups (LPS stress groups) received intraperitoneal (i.p.) LPS injections at a dose of 0.83 mg/kg. This dose was chosen because it elicits a pro-inflammatory cytokine response in the brain, resulting in depressive-like behavior in mice [9, 28]. The control group (CON) was injected (i.p.) with sterile saline. Prior to grouping, any mice displaying a noticeable deviation in their sucrose preference baseline or a significant change in body weight (BW) were excluded from the LPS intervention and subsequent behavioral experiments. The experimental group was randomly divided into four groups: male control (CON-Male), male LPS stress depression group (LPS-Male), female control (CON-Female), and female LPS stress depression group (LPS-Female). Three cohorts of mice were used for metabolomics, quantitative polymerase chain reaction (qPCR), western blotting (WB), and enzyme-linked immunosorbent assay (ELISA) analyses. Following each behavioral assessment, hypothalamus samples were collected for different experiments. Hypothalamus samples from the first cohort were subjected to metabolomics by UHPLC–HRMS/MS and levels of inflammatory cytokine mRNAs and proteins were determined by qPCR and ELISA techniques, respectively (n = 10 mice per group). Hypothalamus samples from the second cohort were used to evaluate the expression levels of essential genes in the steroidogenic pathway by qPCR and to determine the levels of corresponding proteins by WB (n = 10 mice per group, with 6 mice per group used for qPCR and 4 mice per group used for WB). After conducting behavioral assessments on the third cohort of mice, the concentrations of pregnenolone, progesterone, and allopregnanolone in the hypothalamus were determined by ELISA, as per the metabolomics findings. Next, correlation analyses were performed to determine the relationships among the hypothalamic concentrations of pregnenolone, progesterone, and allopregnanolone and the corresponding behavioral data (n = 8 mice/group).

### Behavioral testing and sample collection

The time for the establishment of the LPS-induced depressive-like model was scheduled and the behavioral tests were performed as described previously [5, 28]. Behavioral phenotypes were assessed 24 h after LPS administration during the first 4 h of the dark phase in the light cycle (8:00–12:00 a.m.) under conditions of dim light and low noise.

#### Assessment of sucrose preference and body weight

The sucrose preference test (SPT) has been interpreted as an estimate of anhedonia-like behavior which mimics the inability of patients with depression to experience pleasure from rewarding or enjoyable activities [27, 29]. Anhedonia in rodents is evaluated by SPT based on a two-bottle choice paradigm. Rodents display an innate propensity to consume sweet solutions when provided with a free-choice regimen consisting of both sucrose solution and regular water. Consequently, a decrease in the sucrose preference ratio in experimental mice relative to control mice may be regarded as an indicative metric of anhedonia [29]. To measure the anhedonic effect of LPS treatment, the SPT was conducted as previously described [10]. Briefly, mice were simultaneously presented with two identical bottles, one containing regular water and the other containing sucrose solution (1%, w/v). All mice had access to both water and the sucrose solution for 24 h during a no-stress period. Bottles with water and sucrose solution were switched regularly to control for place preference. The baseline of sucrose preference was recorded thrice during one week. During testing and 24 h after LPS treatment, fluid consumption was measured by weighing the two bottles before and after testing. Sucrose preference was calculated based on the volume of liquid consumed relative to the ingested sucrose solution; that is, sucrose preference = (sucrose intake / [sucrose consumption + water consumption]) × 100%. In addition, body weight was measured before and 24 h after LPS treatment, and changes were analyzed. Changes in the BW of mice were regarded as sensitive indicators of physiological sickness responses.

#### Tail suspension test and forced swimming test

The tail suspension test (TST) and forced swimming test (FST) were employed to detect depressive-like behaviors that simulate feelings of hopelessness in patients with depression. The TST and FST were performed as previously described [9, 10, 26, 30]. The mice were habituated to the behavioral testing room for 30 min. In the TST, each mouse was suspended by its tail using adhesive tape placed 2 cm from the tail tip in an acoustically and visually isolated suspension box. The TST test sessions lasted 6 min, with the last 5 min scored for immobility. Mouse immobility was defined as their passive hanging or complete absence of movement. To assess this, a computerized system (Noldus Inc., Wageningen, Netherlands) processed and analyzed video recordings of each mouse performing the TST. The system determined the duration of immobility by setting a precise threshold level specific to each mouse that would exclude any movement and solely encompass immobility. Time spent below this threshold indicated the duration of immobility. Following completion of the 6-min TST, each mouse was returned to their home cage. They were then transferred to a room in which the FST was conducted. After a 1 h period of acclimatization, each mouse was placed individually in a Plexiglas cylinder (30 cm in height × 15 cm in diameter) filled to 20 cm with water (24 ± 1 °C). The water was changed before each trial. Mice were placed in the water for 6 min and then placed back in their home cages. Throughout the experiment, the mice were recorded and the duration of immobility exhibited during the final 5 min of the test was assessed using the mobility function of the Noldus Observer software (Noldus Inc.). The immobility of a mouse was determined when it ceased struggling and exhibited sluggish movements to maintain buoyancy in the water while ensuring its head remained above the water surface. It has been suggested that prolonged rodent immobility in the FST may indicate a state of helplessness, which can be alleviated by the administration of antidepressant medications. Well-trained observers who were blind to the treatment of each mouse scored the total time spent immobile in the TST and FST.

#### Sample collection

After behavioral assessment, the mice were anaesthetized with 1% sodium pentobarbital (50 mg/kg, i.p.). Once anesthesia was induced [31], transcardially perfused with cold phosphate-buffered saline (PBS, pH = 7.0) was initiated immediately. The whole brain was then rapidly removed and rinsed with ice-cold PBS, and then positioned within a precooled mouse brain mold for sectioning. The hypothalamus was carefully isolated, weighed, then rapidly frozen in liquid nitrogen, and stored at − 80 °C for metabolomic and biochemical analyses. Sample collection was performed collaboratively by very skilled operators to minimize the time taken and to ensure sample quality and homogeneity. After anesthesia, cardiac perfusion and hypothalamus collection took 5–7 min. In the first cohort of mice, the hypothalamus was carefully divided into the left and right hemispheres. The right was used for metabolomics analysis, while the left was used to evaluate cytokine mRNA or protein levels.

### Quantitative real-time PCR

Quantitative real-time PCR (qRT-PCR) was performed to detect gene expression as previously described [9]. Briefly, total RNA was extracted from hypothalamic samples using TRIzol® reagent (Invitrogen Life Technologies, Carlsbad, CA, USA) according to the manufacturer's instructions. cDNA was synthesized from the total RNA using a QuantiNova reverse-transcription kit (QIAGEN). The amplification protocol was 95 °C for 2 min, followed by 40 cycles of 95 °C for 5 s and 60 °C for 10 s using the Applied Biosystems QuantStudio™ Real-Time PCR System (Applied Biosystems Inc., USA). The fold change in mRNA expression was calculated using the comparative cycle method (2^−ΔΔCt^) in the QuantStudio™ Test Development software (version 1.0.3, Applied Biosystems). The primers were designed using BLAST (https://blast.ncbi.nlm.nih.gov/Blast.cgi), synthesized by Sangon Biotech Co., Ltd. (Shanghai, China), and are listed in Supplementary Table S1 (see Additional file 1).

### Enzyme-linked immunosorbent assay

The concentrations of TNF-α, IL-1β, and IL-6 in the hypothalamus were assessed using mouse TNF-α, IL-1β, and IL-6 enzyme-linked immunosorbent assay (ELISA) kits (R&D Systems) following the vendor’s instructions.

### LC–HRMS analysis and identification of metabolic signatures

#### Sample preparation and quality control

Approximately 20 mg of accurately weighed hypothalamus sample was transferred to a 1.5 mL Eppendorf tube and homogenized in a 400 μL mixture of methanol–water (4:1, v/v) with 20 μL 2-chloro-L-phenylalanine (300 mg/mL) as an internal standard. The homogenized mixtures were sonicated in ice water for 10 min, placed at − 20 °C for 30 min, and subsequently centrifuged at 10,000 rpm for 10 min at 4 °C. A 200 μL clear supernatant was transferred to a glass vial and prepared for UPLC–QTOF/MS analysis. Quality control samples were prepared by mixing aliquots of all samples to form a pooled sample, which was analyzed using the same method as the analytical samples. The quality control samples were inserted at regular intervals (every 10 real samples) throughout the analytical run to provide a set of data from which repeatability could be assessed and to monitor the stability of the system and method [32].

#### LC–HRMS analysis

UHPLC–HRMS/MS analysis was performed using a Waters UPLC I-class system coupled with a Waters VION IMS Q-TOF mass spectrometer (Waters, Milford, MA, USA) via an electrospray ionization (ESI) source. The chromatographic and mass spectrometric conditions were optimized to achieve good separation and sensitive analyte detection. Chromatographic separation was performed on Acquity™ UPLC BEH™ C18 column (100 mm × 2.1 mm i.d., 1.7 μm; Waters) using a mobile phase comprising of 0.1% formic acid aqueous solution (A) and acetonitrile containing 0.1% formic acid (B) eluted at a constant flow rate of 0.40 mL/min within 15.5 min of total run time. The gradient elution was programmed as follows: 5–20% B for 0–2 min, linearly increased to 60% B for 2–8 min, 60–100% B for 8–12 min, held constant for 2 min, and finally returned to the initial condition of 5% B over 0.5 min followed by 1 min re-equilibration. The injection volume of all samples was 3.0 µL, and the column temperature was 45 °C. Detection and quantitation were performed in positive and negative ESI sources in full-scan acquisition mode using the following optimized parameters: ion spray voltage, 1.0 kV; capillary temperature, 120 °C; dry gas nitrogen flow rate, 15 L/min; desolvation line temperature, 500 °C; collisional dissociation energy, 6 eV; acquisition range, 50–1000 m/z; acquisition time, 100 ms; and relaxation delay, 20 ms.

#### Data analysis and differential metabolite identification

The acquired raw data were imported into Progenesis QI software (version 2.4; Waters) and processed in successive treatment steps, such as peak alignment, peak picking, and normalization, to obtain the matched and aligned peak data matrix. The peaks of the isotope-labelled internal standards were used to correct the areas of the extracted ion masses. The objective of this analysis was to determine the existence of identifiable ion peaks, to match the peaks, and to determine metabolites that correspond to the identified peaks. Multivariate analyses of the identified metabolites' data matrix were conducted using SIMCA-P software (version 14.1; Umetrics, Umeå, Sweden). Principal component analysis and orthogonal partial least-squares–discriminant analysis (OPLS–DA) were used to visualize the discrimination between different groups. The OPLS–DA model was validated to prevent overfitting using a 300-iteration permutation test. Through the OPLS–DA, metabolites with variable importance in projection (VIP) value > 1.0 were recognized as potentially important metabolites responsible for group discrimination. Two-way analysis of variance (ANOVA) was used to identify significantly altered (*P* < 0.05) metabolites among the four groups. If a significant difference was observed, the Bonferroni's post hoc test was used to determine whether the two groups differed significantly. If a potentially important metabolite was also significantly altered (*P* < 0.05 and VIP > 1.0), it was identified as the key differential metabolite for the groups being compared. The fold-change of the identified differential metabolites (DMs) was calculated as the average mass response (area) ratio between groups.

### Bioinformatic analysis

To verify the composition of the identified DMs in biochemical classification, their biochemical characteristics were investigated by referencing annotations from the Human Metabolome Database (HMDB; http://www.hmdb.ca/), KEGG (http://www.genome.jp/kegg/), LIPID MAPS (http://www.lipidmaps.org) [33], and PubChem (https://pubchem.ncbi.nlm.nih.gov/). In addition, to verify the molecular pathways and explore the biological processes (BPs) of molecules of interest among the identified metabolites, MetaboAnalyst 5.0 (https://www.metaboanalyst.ca/), a comprehensive web-based bioinformatics analysis tool suite for metabolomics [34], was used to link the identified DMs to BP and metabolic pathways, and to visualize the results. The enrichment analysis module and pathway analysis module in MetaboAnalyst 5.0, which are mainly based on the small molecule pathway database, version 2.0 (SMPDB 2.0; http://smpdb.ca/) [35] and the KEGG knowledgebase, were used to identify the significantly altered metabolite sets and metabolic pathways, respectively. “Fisher’s exact test” and “out-degree centrality” were used for the overrepresentation analysis and the pathway topology analysis of the DMs, respectively. To create a heatmap in which the different factors could be compared, the metabolome data of a specific group were normalized to the means and variances of the peak areas of the metabolites by calculating the z-scores. Clustering and heatmap analyses were performed using TBtools software [36].

### Western blotting

The aforementioned bioinformatics findings were validated by western blotting (WB), as described previously [9, 37]. Briefly, hypothalamic tissues were homogenized in precooled lysis buffer (50 mM Tris, pH 7.4, 0.15 M NaCl, 1% Triton X-100, 1% sodium deoxycholate, 0.1% SDS, and 1 mM PMSF). The supernatants were collected, and the total protein concentration was measured using a bicinchoninic acid (BCA) protein assay kit (Thermo Fisher Scientific), according to the manufacturer’s instructions. Equal amounts of protein were separated on a 15% SDS–polyacrylamide gel and then transferred to PVDF membranes (Millipore, USA). After blocking for 1 h at room temperature, transferred membranes were incubated overnight at 4 °C with different primary antibodies, including steroidogenic acute regulatory protein (StAR) antibody (1:1,000; ABclonal, Cat#A16432, RRID: AB_2772418), Cytochrome P450 11A1 (CYP11A1) antibody (1:2,000; Invitrogen, Cat#PA5-37,359, RRID: AB_2554026), CYP11B1 antibody (1:1,000; ABclonal, Cat#A7664, RRID: AB_2769095), CYP11B2 antibody (1:1,000; Invitrogen, Cat# PA5-67,694, RRID: AB_2691688), 5α-reductase type I (SRD5A1) antibody (1:1,000; ABclonal, Cat#A14787, RRID:AB_2761663), and 5α-reductase type II (SRD5A2) antibody (1:1,000; ABclonal, Cat#A19762). GAPDH was detected with an anti-GAPDH antibody (dilution 1:10,000; Proteintech, Cat#60,004–1-Ig, RRID: AB_2107436). After incubation with horseradish peroxidase-conjugated secondary antibodies, the bands were visualized using an ECL kit (Millipore) and analyzed using Bio-Rad Image Lab software. For analysis, protein levels were normalized to GAPDH on the same gel.

Intracerebroventricular cannulation and central pregnenolone administration.

Mice were anaesthetized with isoflurane (3% induction, 1.5% maintenance) and implanted with a cannula (internal diameter, 0.25 mm; RWD, Shenzhen, China) into the third ventricle (coordinates: anterior–posterior, − 1.60 mm; medial–lateral, 0.0 mm; dorsal–ventral, 5.0 mm). Dummy cannulas (RWD) were then inserted into the guides, and the skull was subsequently covered with dental cement. After surgery, the mice were returned to their cages and allowed to recover for at least 7 days before being studied. The third ventricles of cannulated mice were infused with 2.0 μL of either artificial cerebrospinal fluid (ACSF) or pregnenolone (1.0 or 5.0 μg/mL, Sigma-Aldrich) immediately after LPS (i.p.) injections. Following LPS administration, a 24-h SPT was conducted, followed by the reinfusion of pregnenolone into the third ventricle. Subsequently, the FST was performed. This experiment employed 24 female (n = 6 per group) and 24 male (n = 6 per group) mice.

### Pharmacological inhibition of 5α-reductase

Female and male C57BL/6 J mice were subjected to intracerebral stereotaxic injections of the 5α-reductase inhibitor dutasteride (10 μg/μL, MedChemExpress, Cat#HY-13613) or vehicle (20% of 2-hydroxypropyl beta-cyclodextrin in 0.9% NaCl, MedChemExpress, Cat#HY-101103). Dutasteride effectively inhibits 5α-reductase isozymes, as SRD5A1 and SRD5A2, in mice [38]. The mice received bilateral infusions of dutasteride or vehicle into the hypothalamus 2 h prior to LPS administration. Subsequently, behavioral and biochemical tests were performed. This experiment employed 32 female (n = 8 per group) and 36 male (n = 9 per group) mice with suitable baselines for BW and sucrose preference.

### Hypothalamic neurosteroid extraction and quantitation

Hypothalamic tissues were homogenised in 150 μL of acetonitrile, followed by centrifugation at 12000 g for 10 min at 4 °C to separate the supernatants. Subsequently, liquid–liquid extraction was conducted using 300 μL of n-hexane to isolate the neurosteroid fraction from the remaining components of the brain tissue. The homogenates were vigorously shaken for 30 min at room temperature. Next, the two phases were separated, and the neurosteroid fraction was recovered. Subsequently, the samples were subjected to drying in a preheated vacuum at 37 °C using a SpeedVac (Eppendorf). The dried steroid residues were then stored at − 20 °C until they were resuspended in a solution of PBS + 0.1% gelatin + 5% ethanol to quantify pregnenolone, progesterone, and allopregnanolone. Quantification was performed using a validated ELISA (Novus; pregnenolone, Cat#NBP2-68,102; progesterone, Cat#NBP2-60,125; and allopregnanolone, Cat#NBP3-18,698) according to the manufacturer's instructions. The optical densities at 450 nm were compared with a curve generated from standard solutions that had also been diluted in resuspension buffer.

### Statistical analysis

Statistical analyses were performed using GraphPad Prism software (version 9.0; GraphPad Software, San Diego, CA, USA) and data are presented as means ± standard error of the mean (SEM). One-way analyses of variance (ANOVA) was conducted, employing Tukey’s test (assuming equal variance) or Dunnett’s T3 test (not assuming homogeneity of variance), for post-hoc analysis to compare three or more groups. One-way ANOVA was used to examine the impact of pregnenolone infusion or inhibition of 5α-reductase on hypothalamic inflammation and depressive-like behaviors in male or female mice. Two-way ANOVA (LPS *vs.* CON × Female *vs.* Male) was performed for measures of BW loss, depressive-like behaviors in the SPT, TST and FST, levels of cytokines, metabolites, and of key mRNAs and proteins of the steroidogenic pathway. Two-way ANOVA followed by a Bonferroni's post-hoc test for multiple comparisons was used to analyze the possible effects of LPS treatment or sex, if the interaction was significant. Correlations were determined using Pearson’s correlation analysis. The levels of significance are indicated by *P < 0.05, **P < 0.01, and ***P < 0.001. Statistical parameters, including sample size (n), *t/F* value, *P* or adjusted *P* value, and the analysis method used for each experiment are provided in (see Additional file 2: Table S2).

## Results

### Assessing the LPS-induced depressive-like model in mice of both sexes

**Fig. 1:**
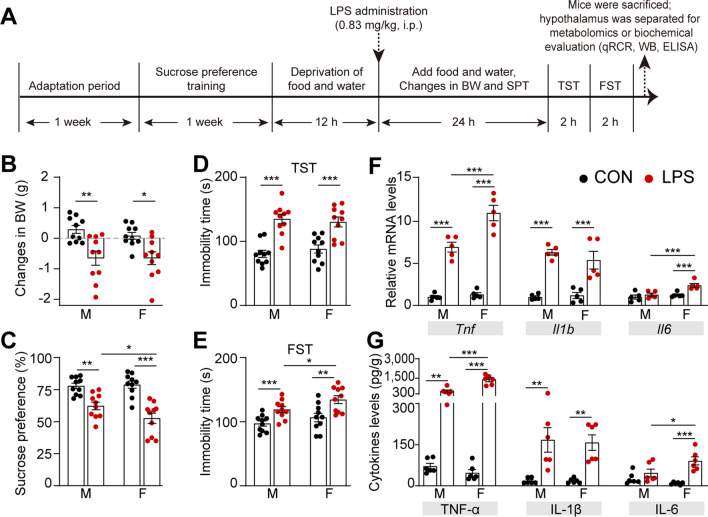
Assessment of the LPS-induced depressive-like behaviors and hypothalamic inflammatory cytokines in female and male mice. **A** Experimental timeline and design. **B**–**E** Body weight changes (**B**) and sucrose preference test (**C**), tail suspension test (**D**), and forced swimming test (**E**) results in female and male mice after LPS exposure, n = 10 mice/group. **F**, **G** The mRNA (**F**) and protein (**G**) levels of hypothalamic cytokines (TNF-α, IL-1β, and IL-6), *n* = 5 (**F**) *n* = 6 (**G**) mice/group. All data are presented as means ± SEM and were analyzed by two-way ANOVA (sex × treatment) followed by Bonferroni's post hoc tests. *P < 0.05, **P < 0.01, ***P < 0.001. *LPS* lipopolysaccharide-treated group, *CON* saline-treated group, *BW* body weight, *SPT* sucrose preference test, *TST* tail suspension test, *FST* forced swimming test

Adult male and female C57BL/6 J mice were challenged with LPS or saline, weighed 24 h later, and then assessed for depressive-like behavior. Mice were then euthanized and their hypothalami collected. Forty mice (n = 10/group) were subjected to hypothalamus metabolomics and to statistical analysis of behavioral changes. The behavioral performance of LPS-related depression in SPT, TST, and FST, was evaluated. The time schedule for inducing depressive-like behavior is depicted in Fig. 1A. To validate the murine depression model, physiological sickness responses were assessed based on the changes in BW. Following LPS exposure, two-way ANOVA revealed a significant main effect of LPS treatment [*F*_(1, 18)_ = 18.870, *P* < 0.001], and no significant interaction between the sex and LPS treatment [*F*_(1, 18)_ = 0.400, *P* = 0.535] (Fig. 1B). Anhedonic-like behavior was evaluated using the SPT, which estimates an animal’s hedonic-seeking behavior by measuring the percentage of sucrose solution consumption. We detected a significant main effect of LPS challenge on the percentage of sucrose consumption [*F*_(1, 18)_ = 38.568, *P* < 0.001] with a close significant interaction between sex and LPS treatment [*F*_(1, 18)_ = 3.865, *P* = 0.065] (Fig. 1C). To assess the impact of inflammatory stress on depressive-like behaviors in both sexes, the TST and FST were employed. The two tests quantify an animal's “efforts” to fight in inescapable situation in the absence of apparent deficits in locomotor deficits. Regarding immobility time in the TST, two-way ANOVA revealed a significant main effect of LPS treatment [*F*_(1, 18)_ = 64.402, *P* < 0.001], and no significant interaction between the sex and LPS treatment [*F*_(1, 18)_ = 0.678, *P* = 0.421] (Fig. 1D). In the FST, a sex-dependent increase in immobility duration was noted in LPS-treated male mice compared to their female counterparts. The results of the two-way ANOVA indicated that the main effect of LPS treatment was significantly evident [*F*_(1, 18)_ = 45.708, *P* < 0.001], as were the main effect of sex [*F*_(1, 18)_ = 6.555, *P* = 0.020] and the interaction between sex and LPS treatment [*F*_(1, 18)_ = 4.446, *P* = 0.049]. Subsequent examination using Bonferroni's post hoc test demonstrated a noteworthy increase in the duration of immobility in male and female mice subjected to LPS compared to their saline-administered counterparts. In contrast to their male counterparts, female mice exhibited a notably increased immobility in the FST (*P* < 0.05, Fig. 1E).

Inflammatory cytokine expression in the hypothalamus.

The assessment of hypothalamic inflammatory responses induced by LPS stress involved the detection of the three inflammatory cytokines TNF-α, IL-1β, and IL-6 that play crucial roles in the pathogenesis of depression [5, 7, 11, 39]. The mRNA and protein levels of these cytokines were analyzed by qRT-PCR and ELISA, respectively. Regarding *Tnf* mRNA levels, a highly significant interaction between LPS treatment and sex was observed [*F*_(1, 8)_ = 31.260, *P* < 0.001], and the main effect of LPS was found to be statistically significant [*F*_(1, 8_) = 120.863, *P* < 0.001], as was the main effect of sex [*F*_(1, 8)_ = 41.985, *P* < 0.001]. A non-significant interaction between LPS treatment and sex was observed in the *Il1b* mRNA level [*F*_(1, 8)_ = 1.735, *P* = 0.224]. Furthermore, the main effect of LPS was statistically significant [*F*_(1, 8)_ = 45.219, *P* < 0.001], whereas the main effect of sex was not significant [*F*_(1, 8)_ = 0.777, *P* = 0.404]. Additional statistical analyses revealed a significant interaction between LPS treatment and sex in relation to the *Il6* mRNA level [*F*_(1, 8)_ = 19.826, *P* = 0.002], as well as significant main effects for LPS [*F*_(1, 8)_ = 20.729,* P* = 0.002] and sex [*F*_(1, 8)_ = 17.091, *P* = 0.003] (Fig. 1F). Akin to transcriptional expression, the protein levels of TNF-α (LPS treatment × Sex: [*F*_(1, 10)_ = 29.129, *P < 0.001], LPS:* [*F*_(1, 10)_ = 90.964, *P* < 0.001], and Sex: [*F*_(1, 10)_ = 26.067, *P* < 0.001]), IL-1β (LPS treatment × Sex: [*F*_(1, 10)_ = 0.027,*** P = 0.874], and LPS:*** [*F*_(1, 10)_ = 42.830, ***P < 0.001]),*** and IL-6 (LPS treatment × Sex: [*F*_(1, 10)_ = 4.647, ***P = 0.057], and LPS:*** [F_(1, 10)_ = 38.286, ***P < 0.001])*** in the hypothalamus demonstrated a consistent response pattern to LPS intervention in male and female mice (Fig. 1G). These findings indicate that the inflammatory response in the hypothalamus was more pronounced in female mice than in male mice when exposed to LPS stress.

### Sex-specific metabolic signatures in the hypothalamus

**Fig. 2:**
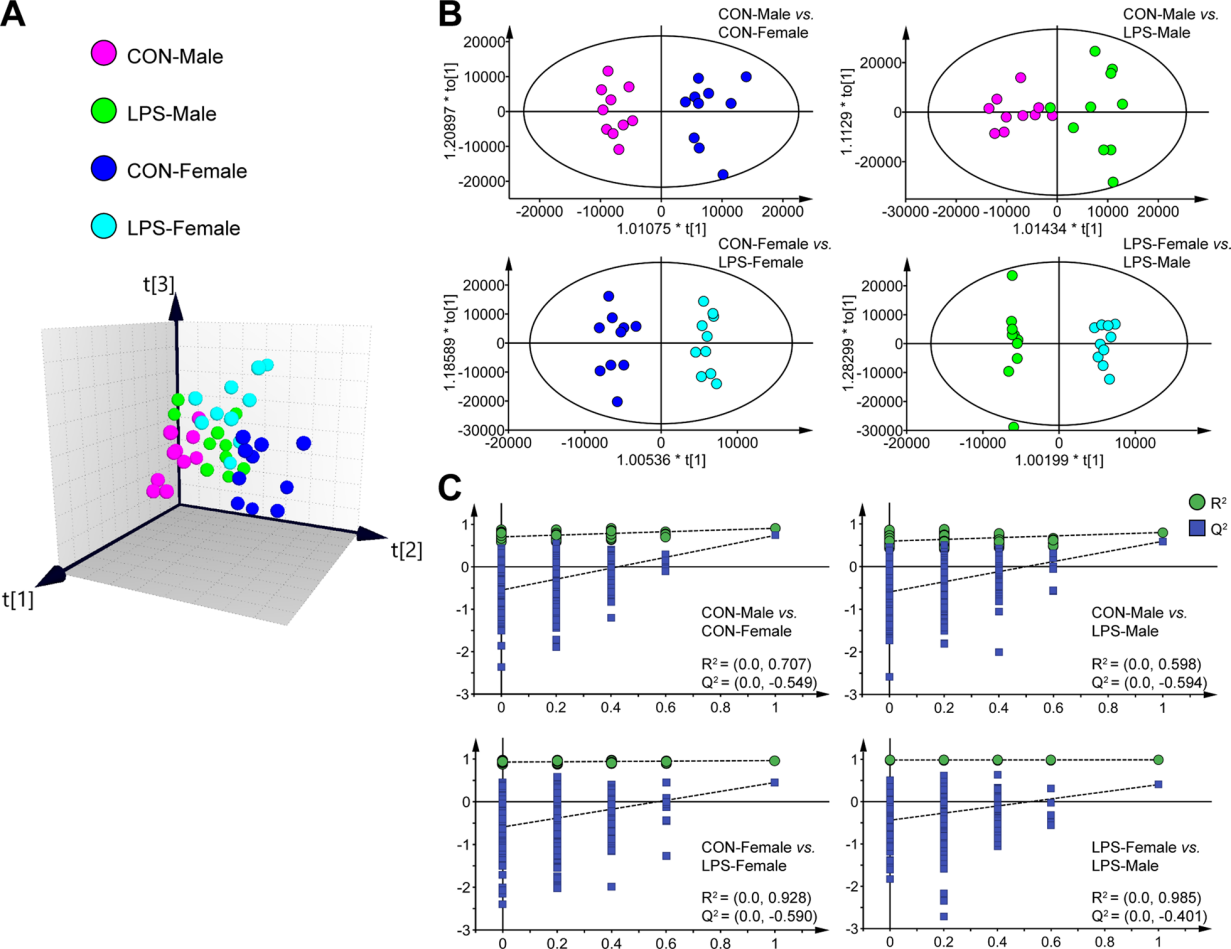
Metabolomics analysis of the hypothalamus from male and female mice with LPS-induced depressive-like behavior compared to their control counterparts. **A** 3D view of the OPLS-DA model showing four clearly separated groups, including saline-treated male controls (CON-Male, purple), LPS-treated depressive-like male mice (LPS-Male, green), saline-treated female controls (CON-Female, blue), and LPS-treated depressive-like female mice (LPS-Female, turquoise). **B** The OPLS–DA model shows a more noticeable separation between t each group pair. The colours correspond to those in (**A**). **C** The permutation test suggests the validity of the OPLS–DA model between each group pair, as the Q^2^ and R^2^ values yielded by the permutation test were higher than their original values

Next, we assessed whether substantial sex differences in the metabolic profiles of the hypothalamus occurred in mice in response to the LPS challenge. To obtain a preliminary understanding of the hypothalamic alterations in the metabolome, we performed a multivariate pattern recognition analysis (Fig. 2). The metabolites detected in the combined positive and negative ESI modes were used to perform OPLS–DA. Initially, the OPLS–DA score plots revealed distinct partitions between male and female control mice (R^2^X = 0.290, R^2^Y = 0.908; Fig. 2B), indicating divergence in hypothalamic metabolic patterns in the absence of inflammation. This finding is consistent with previous reports on sexual dimorphism in the hypothalamus [22, 25]. In addition, the OPLS–DA score plots demonstrated marked partitions between depressive-like mice of both sexes and their healthy counterparts, with minimal overlap (male, R^2^X = 0.463, R^2^Y = 0.801; female, R^2^X = 0.495, R^2^Y = 0.964; Fig. 2B). A notable dissimilarity in metabolic profiles was observed between depressive-like male and female mice (R^2^X = 0.702, R^2^Y = 0.989; Fig. 2B). Specifically, positive R^2^Y and Q^2^Y values indicated robust metabolic differences between groups. Furthermore, the validity of the constructed OPLS–DA models between different comparison groups was determined using 300-iteration permutation tests. The higher original Q^2^ and R^2^ values compared to their corresponding permutated values indicated that the models were not overfitted, thus corroborating the robustness of the results (Fig. 2C). Furthermore, to identify the metabolites responsible for distinguishing sex differences between groups based on scan acquisition modes, we performed independent OPLS–DA analyses of the metabolic data in both positive (ESI +) and negative (ESI–) ion modes. The OPLS–DA outcomes consistently demonstrated clear separations in both detection modes (see Additional file 3: Fig. S1) and indicated that certain DMs detected in the ESI + and ESI– modes contributed to the separations observed in the different groups. A metabolite level difference was deemed significant when the OPLS–DA model yielded a VIP value greater than 1.0 and the two-tailed Student's *t* test resulted in a *P* value of less than 0.05 between the two groups. The findings of this analysis indicated the detection of DMs that were accountable for distinguishing between two experimental groups, namely 121 DMs in CON-Female *vs.* CON-Male, 124 DMs in LPS-Male *vs.* CON-Male, 61 DMs in LPS-Female *vs.* CON-Female, and 37 DMs in LPS-Female *vs.* LPS-Male (see Additional file 3: Fig. S2, Additional file 4: Tables S3–S6).

**Fig. 3:**
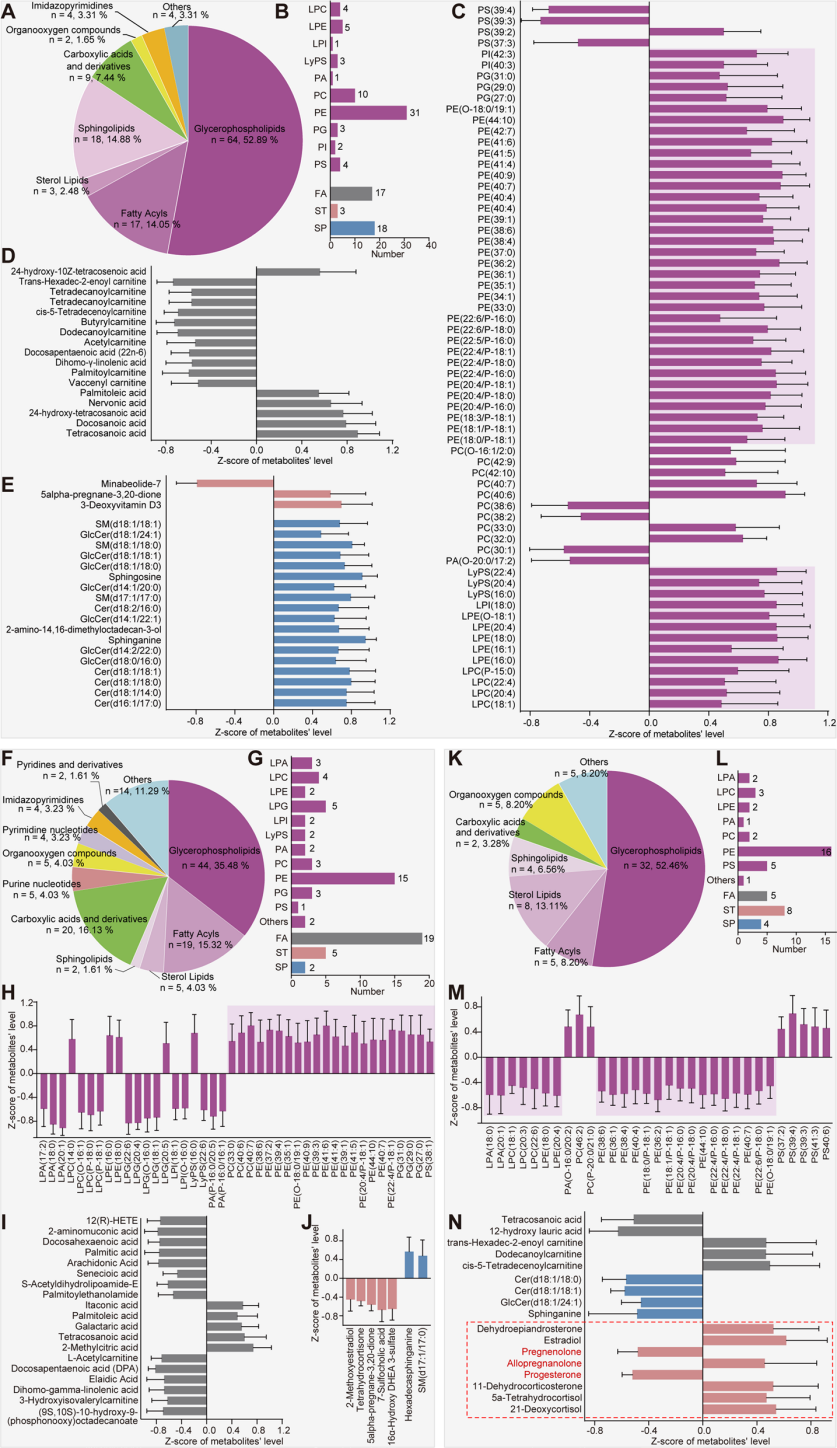
Sex-specific hypothalamic metabolic signatures of male and female mice with LPS-evoked depressive-like behavior. **A**–**E** Hypothalamic metabolic signatures of saline-treated male and female controls. **A** Comprehensive biochemical classification of all identified differential metabolites (DMs) was clustered and visualized as a pie chart. **B** Lipid subgroups and lipid molecule counts according to the annotated lipid classifications of the LIPID MAPS and HMDB databases. **C**–**E** Levels of lipid metabolites belonging to glycerophospholipids (**C**), fatty acyls (**D**), sterol lipids, and sphingolipids (**E**) in CON-Male mice relative to CON-Female mice. **F**–**J** Hypothalamic metabolic signatures in saline- and LPS-treated male mice. **F** Pie chart depicting the biochemical classification of all DMs. **G** Lipid subgroups and lipid molecule counts based on LIPID MAPS and HMDB annotations. **H**–**J** Glycerophospholipids (**H**), fatty acyls (**I**), sterol lipids, and sphingolipids (**J**) levels in the LPS-Male group compared to the CON-Male group. **K**–**N** Hypothalamic metabolic signatures in saline- and LPS-treated female mice. **K** Biochemical classification of all DMs. **L** Lipid subgroups and lipid molecule counts. **M**, **N** Glycerophospholipids (**M**), fatty acyls, sterol lipids and sphingolipids (**N**) in the LPS-Female *vs.* CON-Female. Fold changes in the lipids are expressed as z-scores; a positive value indicates a higher level, whereas a negative value indicates a lower concentration in each group compared to the corresponding control group. *LPA* lysophosphatidic acid, *PA* phosphatidic acid, *LPC* lysophosphatidylcholine, *PC* phosphatidylcholine, *LPE* lysophosphatidylethanolamine, *PE* phosphatidylethanolamine, *LPG* lysophosphatidylglycerol, *PG* phosphatidylglycerol, *LPI* lysophosphatidylinositol, *PI* phosphatidylinositol, *LyPS* lysophosphatidylserine, *PS* phosphatidylserine, *FA* fatty acyls; sterol lipids, ST; and *SP* sphingolipids

As shown in Fig. 2 and (see Additional file 3: Fig. S1), OPLS–DA indicated discernible differences in the metabolic features of the hypothalamus between female and male mice in the absence or presence of any inflammatory response. Consequently, we next focused on further characterizing these dissimilarities. The biochemical features of the DMs were annotated using HMDB, LIPID MAPS, KEGG, and PubChem database. A comprehensive biochemical classification of the DMs was clustered and visualized (Fig. 3). These analyses provided valuable insights into the biological functions and signaling pathways of the identified metabolites in depressive-like male and female mice. The majority (83.47%) of the 121 DMs differing between the CON-Female and CON-Male groups were lipids and lipid-like molecules [33], including 64 glycerophospholipids (GPs), 17 fatty acyls (FAs), 2 sterol lipids (STs), and 18 sphingolipids (SPs). The remaining DMs were carboxylic acids and their derivatives, organooxygen compounds, and imidazopyrimidines (Fig. 3A). These results indicated that the hypothalamic metabolic signatures that exhibited significant differences were related to lipid metabolism. To gain a more precise understanding of these variations, a more detailed categorisation of lipids and lipid-like molecules was performed (Fig. 3B–E). Based on annotations found in the LIPID MAPS database, 64 distinct GP molecules were categorized into 10 subclasses: lysophosphatidylcholine (LPC), lysophosphatidylethanolamine (LPE), lysophosphatidylinositol (LPI), lysophosphatidylserine (LyPS), phosphatidic acid (PA), phosphatidylcholine (PC), phosphatidylethanolamine (PE), phosphatidylglycerol (PG), phosphatidylinositol (PI), and phosphatidylserine (PS; Fig. 3B, purple column). PE constituted nearly 50% of GP molecules, exhibiting a uniform trend of elevated expression with positive z-scores in the hypothalamus of CON-Female mice compared to the CON-Male cohort. This trend is also evident in PI, PG, LyPS, LPI, LPE, and LPC. Conversely, only seven GP metabolites were detected at low levels in the hypothalamus of CON-Female mice (Fig. 3C). Furthermore, the CON-Female and CON-Male groups were distinguished by 17 FA metabolites (11 down- and 6 up-regulated, Fig. 3D), 18 SPs (all upregulated), and 3 STs (1 down- and 2 upregulated, Fig. 3E).

**Fig. 4:**
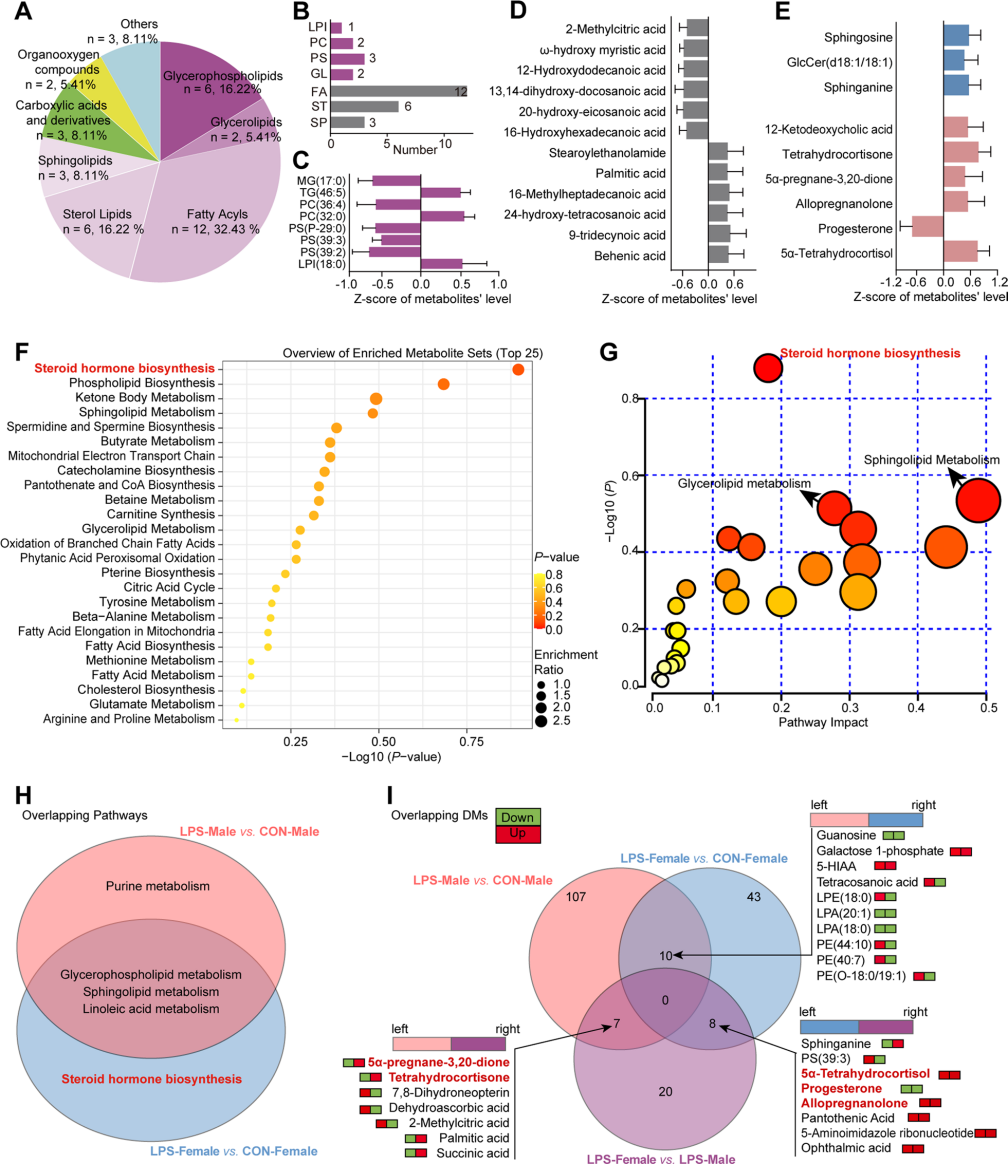
The hypothalamic steroid hormone biosynthesis is significantly affected by inflammation, particularly in female mice. **A**–**E** Hypothalamic metabolic signatures of LPS-treated female and male mice. **A** Biochemical classifications of all DMs. **B** Lipid subgroups and lipid molecule counts. **C**–**E** Glycerophospholipids (**C**), fatty acyls (**D**), sterols, and sphingolipids (**E**) in LPS-Female *vs.* LPS-Male. Relative levels of the lipids by z-scores, a positive value indicates a higher level, whereas a negative value indicates a lower concentration in the LPS-Female group. **F**, **G** Implications of DMs in biological processes (**F**) and metabolic pathways (**G**) in the LPS-Female *vs.* LPS-Male comparison based on MetaboAnalyst 5.0 analyses. **H** Venn plot of significantly affected metabolic pathways in LPS-treated female and male mice. Perturbations of steroid hormone biosynthesis are apparent in the hypothalamus of the female depression model. **I** Venn diagram of DMs from different comparisons. Red boxes represent increased levels, and green boxes represent decreased levels. *FA* fatty acyls, *LPG* lysophosphatidylglycerol, *LPI* lysophosphatidylinositol, *MG* monoacylglycerol, *PC* phosphatidylcholine, *PG* phosphatidylglycerol, *PS* phosphatidylserine, *SP* sphingolipid, *ST* sterol lipid, *TG* triacylglycerol

Given sex-specific modifications in lipid metabolism in the plasma and brain during ageing and dietary variations [40, 41], it is reasonable to hypothesize that dissimilarities in hypothalamic lipid metabolism under physiological conditions (Fig. 3A–E) may serve as the underlying basis for sex-specific manner of mood alterations or psychiatric disorders in response to inflammatory stimuli. Consequently, additional analyses were conducted to disclose the lipid metabolism traits in the hypothalamus of male and female mice in the presence of inflammatory stress (Fig. 3F–N). Seventy metabolites differentially expressed in the LPS-Male group compared to the CON-Male group were classified as lipids and lipid-like molecules, comprising over 50% of all DMs (Fig. 3F). The profile of the lipid-like molecules consisted of 70 compounds, including 44 GPs, 19 FAs, 5 STs, and 2 SPs (Fig. 3G–J). Within the GP subclass, it was observed that 15 PE metabolites were the most prevalent subgroup and significantly increased in the LPS-Male group compared to the CON-Male group. The levels of three PC (PC 40:7, PC 40:6, and PC 33:0), three PG (PG 29:0, PG 27:0, and PG 31:0), one PS (PS 38:1), and two LPE (LPE 16:0 and LPE 18:0) metabolites were elevated, whereas those of three lysophosphatidic acid (LPA), two LPE, two LPI, and two PA metabolites were decreased (Fig. 3H). Similarly, the hypothalamus of LPS-treated male mice showed significant modifications in the FA subclass compared to their CON-Male counterparts, as evidenced by a reduction in the levels of 14 and an elevation in the levels of 5 FA species (Fig. 3I). Among the ST and SP subclasses, five ST metabolites had reduced concentrations, whereas two SP metabolites had elevated levels (Fig. 3J). Likewise, DMs detected in LPS-treated female depressive-like mice, as opposed to their sex-matched controls, were primarily composed of lipid species (> 70%), specifically 32 GPs, 5 FAs, 4 SPs, and 8 STs (Fig. 3K–N). Within the GP subclass, reduced levels were observed in 2 LPA, 3 LPC, 2 LPE, and 16 PE isoforms, whereas increases were noted in 3 PC and 5 PS isoforms (Fig. 3M). Notably, a comparison of female depressive-like mice and their sex-matched controls demonstrated a decrease in the quantity of DMs within the FA subclasses and an increase in the number of ST species (Fig. 3N), in contrast to the other two comparison groups (Fig. 3D, E, in CON-Female *vs.* CON-Male, and Fig. 3I, J, in LPS-Male *vs.* CON-Male). Interestingly, the fluctuation patterns of the disclosed PE isoforms, as depicted in Fig. 3C, H and M, led to a thorough categorization of their individual levels. Distinct expression patterns in PE isoforms were observed, with female mice exhibiting a significant increase, no difference, or a significant decrease in hypothalamic PE metabolites in the presence of neuroinflammation. In contrast, male mice generally showed higher levels of PE isoforms in the hypothalamus under inflammatory conditions (see Additional file 3: Fig. S3). Importantly, 37 DMs in LPS-treated female and male depressive-like mice revealed that the majority (> 75%, 29 lipids) were lipid species. Specifically, these DMs consisted of 6 GPs, 2 glycerolipids (GLs), 12 FAs, 3 SPs, and 6 STs (Fig. 4A, B). The results indicated that a decrease in the proportion of GP species in the DMs, while increases were observed in 12 FA and 6 ST metabolites (Fig. 4C–E), in comparison to the three comparisons illustrated in Fig. 3. It suggested that the difference in hypothalamic metabolism between female and male mice with depressive-like behaviors is not primarily attributable to GPs involved lipid metabolism signaling, but rather to metabolic fluctuations that may be dominated by FA or ST lipids (Fig. 4A–E). Collectively, these data imply that the dissimilarity in the hypothalamic lipid metabolism response to inflammatory stimuli may be involved in sex-specific susceptibility to depression in this mouse model.

### Perturbation of the steroidogenic pathway occurs in the hypothalamus of female mice with inflammation-evoked depression

To verify the BP and molecular pathways among the DMs, a comprehensive metabolic network was constructed using the MetaboAnalyst 5.0. Enrichment analyses were conducted on the enriched metabolite sets derived from various comparison groups to determine the presence of differential metabolite sets associated with specific BPs in the presence or absence of inflammation. Most DMs, as lipid molecules, have a strong association with the primary BPs and lipid metabolism. Specifically, the most prominent BPs were found to be linked to lipid molecules, such as glycerophospholipid metabolism, glycerolipid metabolism, phospholipid biosynthesis, sphingolipid metabolism, and steroid hormone biosynthesis. This suggests a strong association between hypothalamic lipid metabolism and sex disparities (Fig. 4F and see Additional file 3: Fig. S4A–S4C). Pathway analysis is an extension of the BP enrichment analysis of differential metabolite sets to obtain a better understanding of the pathways involved. We identified several pathways that may be aberrant in neuroinflammation-induced female and male depressive-like mice (Fig. 4G and see Additional file 3: Fig. S4D–S4F). In the absence of inflammation, the hypothalamus of healthy male and female mice exhibited distinct metabolic pathways for amino acids (alanine–aspartate–glutamate and purine metabolism) and lipids (glycerophospholipid and sphingolipid metabolism; see Additional file 3: Fig. S4D). In the context of neuroinflammation, metabolic perturbations in the hypothalamus of male depression model mice compared to control male littermates were primarily associated with glycerophospholipid and purine metabolism (see Additional file 3: Fig. S4E). The top three pathways identified in the hypothalamus of female depression model mice were glycerophospholipid metabolism, steroid hormone biosynthesis, and sphingolipid metabolism (see Additional file 3: Fig. S4F). The highest-ranked pathway was the same in female and male model groups compared to their sex-matched healthy controls, i.e., glycerophospholipid metabolism. Purine metabolism and steroid hormone biosynthesis ranked second in the male and female depression model groups, respectively. In our previous study, we verified that purine metabolism is a key hypothalamic pathway involved in male mice with LPS-induced depression [26]. Compared with male depressive-like mice, hypothalamic steroid biosynthesis was the most significantly affected pathway in female depressive-like mice (Fig. 4F–H). We next investigated common and discriminating metabolite expression patterns among the study groups comparing LPS-Male with CON-Male, LPS-Female with CON-Female, and LPS-Female with LPS-Male mice. As shown in Fig. 4I, none of the DMs were common in all three comparisons. The intersection of 10 DMs between LPS-Male vs. CON-Male and LPS-Female vs. CON-Female consisted of three non-lipid molecules and seven GP molecules: tetracosanoic acid, LPE 18:0, LPA 20:1, LPA 18:0, PE 44:10, PE 40:7, and PE O-18:0/19:1. The levels of tetracosanoic acid, LPE 18:0, PE 44:10, PE 40:7 and PE O-18:0/19:1 were increased in LPS-Male mice and decreased in LPS-Female mice relative to their sex-matched controls. In addition, 15 DMs were present in two other comparisons (7 in the intersection between LPS-Male vs. CON-Male and LPS-Female vs. LPS-Male; 8 in the intersection between LPS-Female vs. CON-Female and LPS-Female vs. LPS-Male). These 15 DMs, of which the five ST metabolites 5alpha-pregnane-3,20-dione (5α-DHP), tetrahydrocortisone, progesterone, allopregnanolone, and 5α-tetrahydrocortisol (Fig. 4I) are involved in steroid biosynthesis, are considered important neuroactive steroids [42, 43]. The results obtained from the DM pathway analyses suggest that the steroidogenic pathway of the hypothalamus exhibits sex specificity in the context of depression induced by inflammatory stress, leading to female proclivity.

**Fig. 5:**
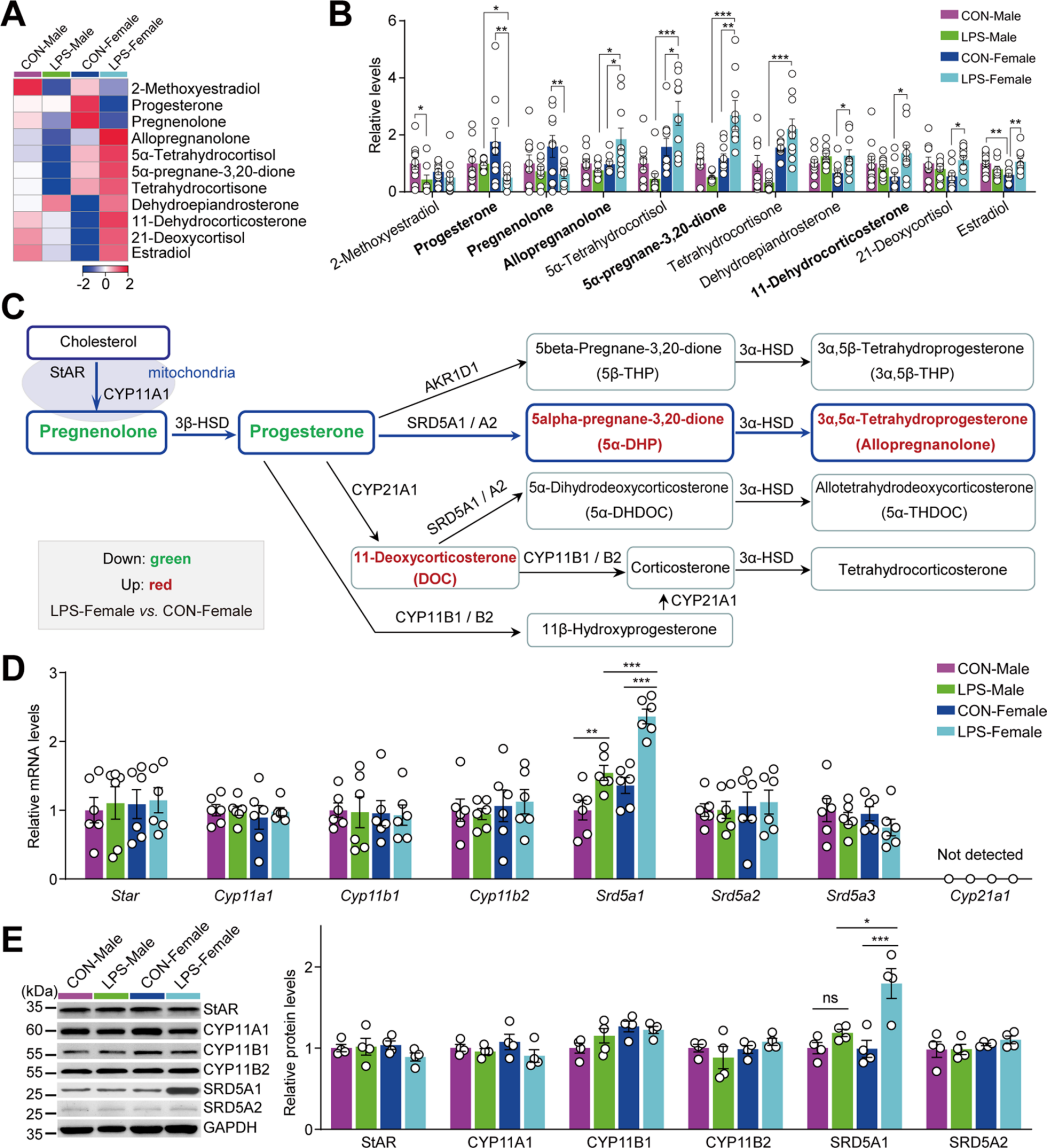
Verification of perturbations in steroidogenic pathways in the hypothalamus of LPS-induced depressive-like female mice. **A**, **B** Heatmap (**A**) and scatter plot (**B**) of the detected steroid metabolites in the hypothalamus of LPS-induced depressive-like mice and sex-matched controls. **A** Raw measurement mean was converted into z-scores, with downregulation denoted by blue, and upregulation indicated by red. **B** Relative levels of steroid metabolites were determined by comparing them to the raw measurement mean of the CON-Male group. **C** Metabolite–protein interaction network of the steroid biosynthesis pathway, according to the KEGG annotations. Differential steroids between LPS-Female and CON-Female mice are labelled with red (increased) and green (decreased) in the network, and essential proteins and unchanged steroid metabolites are designated. **D**,** E** mRNA transcriptional (**D**) and protein (**E**) levels of the key proteins involved in steroidogenic pathways, *n* = 6 (**D**) and *n* = 4 (**E**) mice/group. All data are presented as means ± SEM and were analyzed by two-way ANOVA followed by Bonferroni's post hoc tests. *P < 0.05, **P < 0.01, ***P < 0.001

The hypothalamus is not only a target area for neuroactive steroids, but also a steroidogenic region [44]. Interactions between neurosteroid production and action have been implicated in the emergence of various neuropsychiatric disorders, such as depression, anxiety, and schizophrenia [45]. However, the role of the hypothalamic steroidogenic changes in inflammation-associated depression remains unclear. To fully understand the alterations occurring within the hypothalamic steroidogenic pathways in response to inflammatory stress, the metabolites associated with these pathways were analyzed separately and in detail (Fig. 5A, B). Pregnenolone is considered a vital precursor of all steroid hormones and is related several different metabolic pathways [43, 44, 46], including the synthesis of corticosteroids (cortisol and cortisone), neuroactive steroids (particularly progesterone, another precursor of virtually all steroid hormones), and its metabolism into its reduced derivatives, such as 5α-DHP, allopregnanolone, and 11-deoxycorticosterone. The levels of pregnenolone and progesterone decreased, whereas 5α-DHP, allopregnanolone, and 11-deoxycorticosterone concentrations in LPS-Female mice increased compared to those in CON-Female mice (Fig. 5B). Pregnenolone, progesterone, and allopregnanolone levels in the hypothalamus were validated using ELISA (see Additional file 3: Fig. S5A). Subsequently, referring to KEGG annotations, the altered key metabolic molecules were designated within the steroidogenic pathway, and the essential enzymes implicated in this pathway are shown in Fig. 5C. Neurosteroids are steroid hormone derivatives locally synthesized from cholesterol in the brain. In this process, the steroidogenic acute regulatory protein (StAR, encoded by *Star*) mediates the trafficking of cholesterol to the mitochondria, which is the rate-limiting step in neurosteroid production, and it is subsequently metabolized by cytochrome P450 11A1 (CYP11A1, encoded by *Cyp11a1*) into pregnenolone [44]. Moreover, the enzymatic mechanisms implicated in the conversion of pregnenolone to neuroactive metabolites such as progesterone, 5α-DHP, and allopregnanolone, require the participation of several essential enzymes, namely cytochrome P450 species (CYP21A1, CYP11B1, and CYP11B2), as well as 5α-reductase type I, II, and III isozymes (SRD5A1, SRD5A2, and SRD5A3). The mRNA and protein levels of the associated key enzymes were verified using RT-PCR and WB, respectively. In the presence of inflammatory stress, two-way ANOVA revealed that hypothalamic mRNA [*F*_(1, 20)_ = 41.787, *P* < 0.001] and protein [*F*_(1, 12)_ = 11.996, *P* = 0.005] levels of SRD5A1 exhibited significant changes in both male and female mice, with a more pronounced increase observed in female mice. Bonferroni's post hoc analysis demonstrated a significant increase in the mRNA and protein levels of SRD5A1 in LPS-Female mice compared to LPS-Male mice (Fig. 5D, E). Collectively, these results indicate that the steroid biosynthesis pathway in the hypothalamus of female mice is perturbed by inflammatory stress.

### Central pregnenolone delivery reverses hypothalamic inflammation and depressive-like behaviors in female mice

**Fig. 6:**
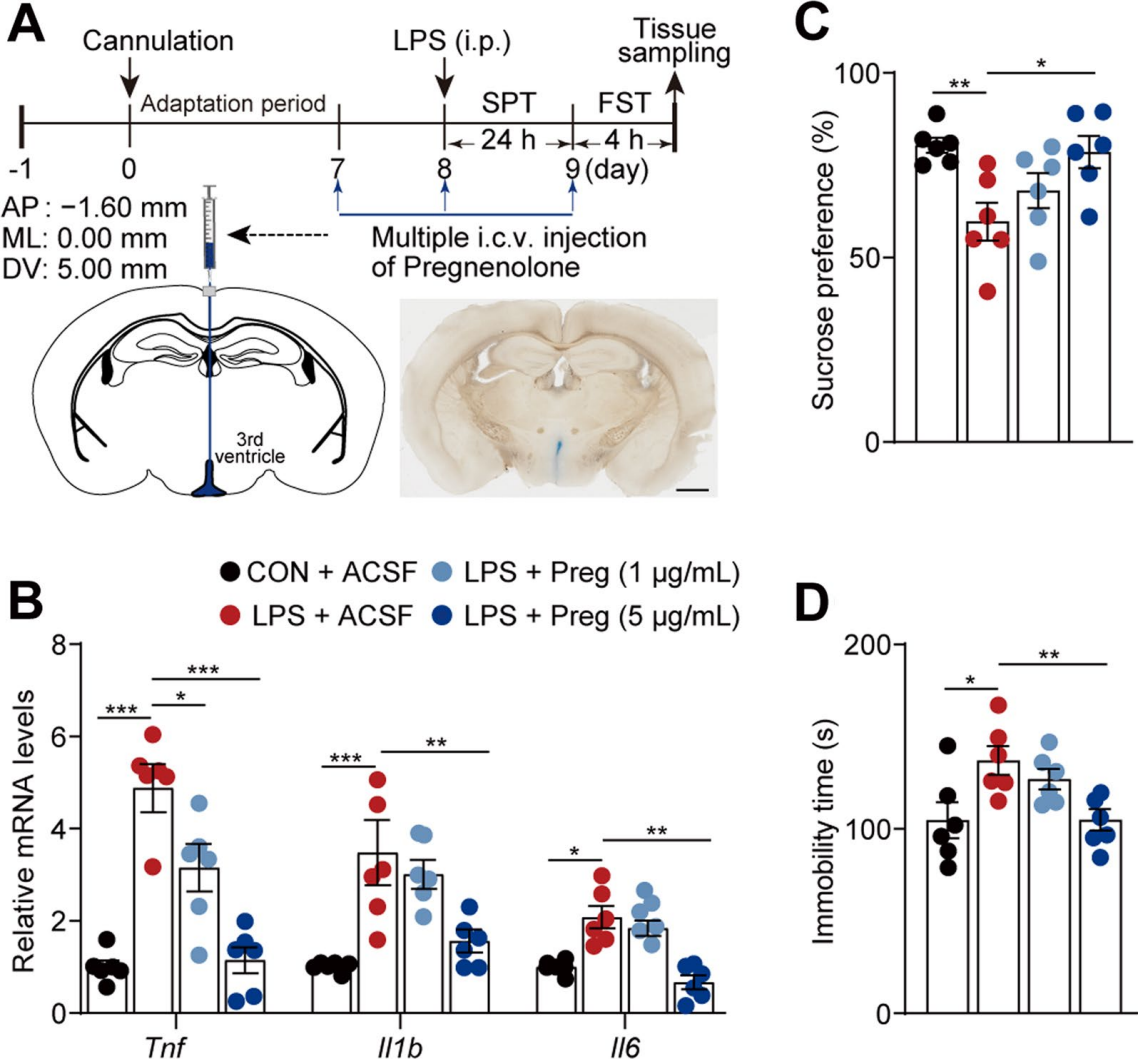
Pregnenolone supplements reversed hypothalamic inflammation and depressive-like behaviors in female mice. **A** Schematic illustration of the central pregnenolone (Preg) delivery and subsequent behavioral tests in female mice. Blue arrows show the injection phases. Schematic representation of the injection site in the third ventricle stained with Evans blue (bottom right, Scale bar 2 mm). **B** Transcriptional levels of hypothalamic *Tnf*, *Il1b*, and *Il6*, after i.c.v. administration of artificial cerebrospinal fluid (ACSF) or pregnenolone (Preg), n = 6 mice/group. **C**, **D** Recorded parameters to assess sucrose preference in the SPT (**C**) and immobility in the FST (**D**) after i.c.v. administration of ACSF or pregnenolone, n = 6 mice/group. All data are presented as means ± SEM and were analyzed using one-way ANOVA with Bonferroni's post hoc tests. *P < 0.05, **P < 0.01, ***P < 0.001. *AP* anterior–posterior, *ML* medial–lateral, *DV* dorsal–ventral, *SPT* sucrose preference test, *FST* forced swimming test; i.c.v., intracerebroventricular

Inflammation plays a critical role in modulating the hypothalamic neurosteroid metabolic pathway, thereby increasing susceptibility to depression. Consequently, the regulation of inflammation could potentially yield comprehensive therapeutic advantages for individuals with depression [2, 5]. The neurosteroid pregnenolone, a crucial precursor of steroid hormones, suppresses inflammation [46] and exhibits potential antidepressant properties [45]. Indeed, the levels of pregnenolone were reduced in the LPS-Female group (Fig. 5B and see Additional file 3: Fig. S5A) and were more significantly correlated with depressive-like behaviors in female mice (see Additional file 3: Fig. S5B–S5G). To ascertain the potential effects of pregnenolone supplements on hypothalamic inflammation and their potential to ameliorate depressive-like behaviors, female mice received pregnenolone via intracerebroventricular (i.c.v.) administration (Fig. 6A). The administration of pregnenolone successfully counteracted the LPS-induced increases in the levels of the pro-inflammatory cytokine mRNAs in female mice (*Tnf*, [*F* = 21.267, *P* < 0.001]; *Il1b*, [*F* = 8.454, *P* < 0.001]; and *Il6*, [*F*_(1, 8)_ = 9.194, *P* < 0.001]; Fig. 6B). As anticipated, the evaluation of the effects of pregnenolone on depressive-like behaviors revealed that pregnenolone treatment (i.c.v.) effectively reversed the diminished sucrose preference ([*F* = 5.323, *P* = 0.007], Fig. 6C) and extended immobility ([*F* = 5.983, *P* = 0.004], Fig. 6D) following LPS intervention. We also investigated the effect of pregnenolone on hypothalamic inflammation and depressive-like behaviors in male mice and found that pregnenolone reduced in the elevated levels of cytokine mRNAs, particularly *Tnf* ([*F* = 9.881, *P* < 0.001]) and *Il1b* ([*F* = 4.769, *P* = 0.012]) induced by LPS. However, it failed to mitigate the depressive-like behaviors induced by LPS. (see Additional file 3: Fig. S6A–C). Taken together, these results clearly indicate that maintaining homeostasis in the neurosteroid biosynthesis pathway is crucial for the hypothalamus of female mice. Pregnenolone supplementation may potentially support homeostasis, thereby mitigating excessive inflammation and improving depressive symptoms in female mice.

### Pharmacological inhibition of 5α-reductase mimics the effects of central pregnenolone delivery in female mice

**Fig. 7:**
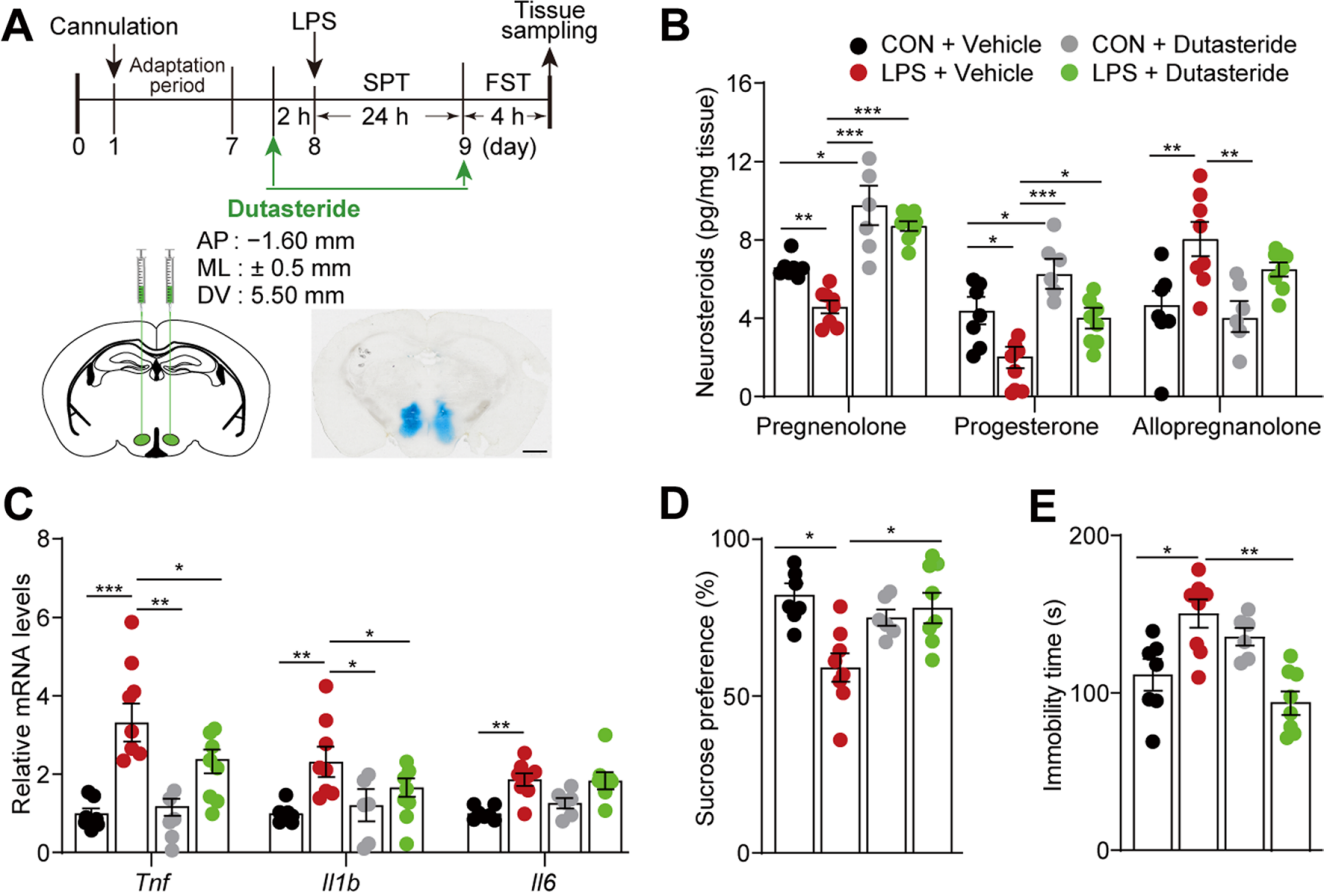
Inhibition of 5α-reductase attenuates hypothalamic inflammation and depressive-like behaviors in female mice. **A** Schematic illustration of dutasteride (a 5α-reductase inhibitor) or vehicle administration via bilateral infusion into the hypothalamus 2 h before LPS injection and subsequent behavioral and biochemical tests in female mice. Schematic representation of the injection site in the hypothalamus stained with Evans blue (bottom right, Scale bar 2 mm). **B** Hypothalamic levels of the three neurosteroids, pregnenolone, progesterone, and allopregnanolone, in female mice, n = 6–8 mice/group. **C** Transcriptional levels of hypothalamic *Tnf*, *Il1b*, and *Il6* expression, *n* = 6–8 mice/group. **D**, **E** Behavioral assessments. Sucrose preference (**D**) and immobility in the FST (**E**), *n* = 6–8 mice/group. All data are presented as means ± SEM and were analyzed using one-way ANOVA with Bonferroni's post hoc tests. *P < 0.05, **P < 0.01, ***P < 0.001

The neurosteroid biosynthesis pathway in the hypothalamus of female mice exhibited a noticeable disruption in response to inflammatory stress, characterized by decreased pregnenolone and progesterone levels, and increased 5α-DHP and allopregnanolone levels (Fig. 5B and see Additional file 3: Fig. S5A). Furthermore, SRD5A1 was upregulated in the hypothalamus of LPS-treated female mice (Fig. 5E). In the brain, the rate-limiting enzyme SRD5A1 is involved in neurosteroidogenesis, particularly the conversion from progesterone to 5α-DHP and allopregnanolone [38]. These findings suggest that rectifying the imbalance within the steroidogenic pathway may be a viable therapeutic strategy for neuroinflammation-associated depression. To test this hypothesis, dutasteride, a 5α-reductase inhibitor capable of inhibiting SRD5A1 [47], was stereotactically injected into the hypothalamus of female and male mice (Fig. 7A). As anticipated, inhibition of 5α-reductase effectively corrected the decrease in pregnenolone and progesterone levels in the hypothalamus of female mice during neuroinflammation. Two consecutive dutasteride administrations significantly sustained elevated levels of pregnenolone, which were approximately twofold higher than those in vehicle-treated mice that underwent LPS challenge (pregnenolone, [*F* = 14.521, *P* < 0.001]; progesterone, [*F* = 11.181, *P* < 0.001]; and allopregnanolone, [*F* = 6.704, *P* = 0.002]; Fig. 7B). Furthermore, administration of the pharmacological inhibitor targeting SRD5A1 effectively mitigated the LPS-induced elevated transcriptional expression of pro-inflammatory cytokines in female mice (*Tnf*, [*F* = 7.430, *P* = 0.001]; *Il1b*, [*F* = 4.958, *P* = 0.008]; and *Il6*, [*F* = 6.481, *P* = 0.002]; Fig. 7C). In addition, this intervention reversed the LPS-induced reduction in sucrose preference ([*F* = 4.628, *P* = 0.010], Fig. 7D) and the prolonged immobility ([*F* = 5.967, *P* = 0.003], Fig. 7E) induced by LPS. Nevertheless, administration of dutasteride did not effectively mitigate the hypothalamic inflammation or the depressive-like behaviors triggered by LPS in male mice (see Additional file 3: Fig. S6D–S6G). These findings suggest that the pharmacological inhibition of 5α-reductase mimics the effects of central pregnenolone infusions in female mice.

## Discussion

This study reports a key mechanism by which the hypothalamic steroidogenic pathway mediates susceptibility to inflammation-induced depression in female, but not male mice. Our findings indicated that the LPS intervention triggered neuroinflammation and induced depressive-like behaviors in male and female mice. Furthermore, disturbances in hypothalamic lipid metabolism, including glycerolipid metabolism, glycerophospholipid metabolism, phospholipid biosynthesis, and sphingolipid metabolism, were observed in both sexes. Notably, the neurosteroid biosynthesis pathway in the hypothalamus of female mice experienced considerable alterations during inflammatory stress, characterized by a reduction in pregnenolone and progesterone levels and an increase in 5α-DHP and allopregnanolone concentrations. The i.c.v. administration of pregnenolone or inhibition of hypothalamic SRD5A1 alleviated the excessive neuroinflammatory state and depressive symptoms in female mice by correcting the imbalance in the steroidogenic pathway. Considering the specificity of this pathway, these findings suggest that maintaining homeostasis of the steroidogenic pathway is a potential therapy for neuroinflammation-related depression, especially in females with depression.

Inflammation appears to have a greater depressogenic effect in women than on men [17, 19, 39]. Converging evidence from both animal and human studies substantiates the existence of sex-based disparities in the immune response to acute stressors or activation of the HPA axis [8, 17]. Recent studies have examined the manifestations of depressive-like characteristics in rodents of both sexes subjected to inflammation-induced stress [8, 39]. Our results corroborate these findings by demonstrating that female mice exhibit more pronounced symptoms of depression following the LPS challenge. Moreover, we demonstrated that molecular signals may determine sex differences in neuroinflammation-related depression. Adzic et al*.* also observed activation of the HPA axis in both male and female Wistar rats with depressive-like behaviors induced by LPS and described sex-specific alterations in hypothalamic molecular signaling. In male rats, the activation was related to the retention of glucocorticoid receptors, whereas in female rats it was related to diminished neurotrophic capacity [8]. Mello et al*.* observed increased lipid peroxidation in the hypothalamus of female, but not male, Swiss mice following LPS exposure [39]. Furthermore, we have previously observed disturbances in purine metabolism, ephrin receptor signaling, and glutamatergic transmission were observed in the hypothalamus of male CD-1 mice with depressive-like behaviors induced by LPS [9, 26]. In the present study, perturbation of purine metabolism and glycerophospholipid metabolism occurred primarily in the hypothalamus of male C57BL/6 J mice with depressive-like behaviors compared to control male littermates. Conversely, the perturbation of the steroidogenic pathway occurs in the hypothalamus of female mice with inflammation-evoked depression. This suggests that differences in the genetic background and hypothalamic metabolic responses to inflammatory stress may contribute to the sex-specific nature of depression. More importantly, the hypothalamic transcriptome and neuroendocrine discrepancies between males and females reflect their sexual dimorphism [8, 13, 23], which mediates the prominent sex-dependent disparity in neuroimmune responses [17], behaviors [14], and antidepressant efficacies [25]. In addition, the sex-specific abnormalities in lipid metabolism were not only recorded in the hypothalamus and plasma during normal ageing [40, 48, 49] but have also been considered as a bridge between neuroinflammation and depression [10, 11, 50, 51]. This indicates that discrepancies in hypothalamic lipid metabolism may potentially contribute to a female predilection for depression. The hypothalamic metabolomics results of the current study support this hypothesis and reveal a remarkable differentiation in lipid metabolism in the hypothalamus between female and male mice in the absence or presence of inflammatory stimuli.

Lipids encompass a collection of intricate compounds that serve as fundamental constituents for crucial structures like cell membranes and myelin sheaths, regulate intercellular communication between brain cells, and potentially influence brain function [40, 49, 51]. Different lipids are transformed into one another via metabolic catalysis. For instance, fatty acyl substituents can contribute to the composition of complex lipids such as glycerolipids, glycerophospholipids, sphingolipids, sterol lipids, and eicosanoids [49, 52, 53]. Under conditions of stress or physiological ageing, variations in lipid metabolism occur, leading to potential disruptions in lipid turnover and subsequent breakdown of distinct lipid metabolic pathways [48, 49, 51]. This is also supported by our data. First, metabolomics analysis showed that lipids and lipid-like molecules accounted for the majority of DMs. Second, distinct expression patterns of hypothalamic PE metabolites were observed in female mice, with male animals exhibiting the main augmentation under neuroinflammation. Finally, pathway analysis revealed abnormalities in glycerophospholipid, sphingolipid, and linoleic acid in both male and female mice. Notably, female mice displayed distinct modifications in the neurosteroid biosynthesis pathway, whereas male mice exhibited specific alterations in purine metabolism, as previously documented [26]. Moreover, sexual disparities in the production of neuroactive steroids and in 5α‐reductase (especially SRD5A1) levels within the hypothalamus following the LPS challenge were observed. Collectively, these findings imply that the heterogeneity and interconversion of lipids in lipid metabolism pathways in response to inflammatory stimuli serve as fundamental determinants of the disparity in susceptibility to depression between male and female mice. After conducting bioinformatics analysis and molecular validation, our attention was ultimately directed towards sex-specific changes in the hypothalamic neurosteroid metabolism pathway of female mice with inflammation-evoked depression.

Although evidence supporting the involvement of neurosteroids in rodent models of psychiatric disorders is compelling [45, 53, 54], the potential role of neuroactive steroids in mediating sex differences in vulnerability to depression, as well as variations in neurosteroidogenesis contributing to the divergence of sex-specific responses of the HPA axis to stress, have yet to be thoroughly investigated. The primary objective of our study was to examine this issue. Dysregulation of hypothalamic neurosteroids and their derivatives, particularly pregnenolone, progesterone, and allopregnanolone, has been observed in female mice due to inflammatory stress. Indeed, social stress and inflammatory induce abnormal alterations in the levels of these neurosteroids, which mediate the occurrence and progression of mental illnesses [46, 52, 53]. Our results revealed a decrease in pregnenolone and progesterone levels, as well as an increase in 5α-DHP and allopregnanolone levels, specifically in the hypothalamus of females, but not males. Among these neurosteroids, pregnenolone was the most correlation to behavioral changes in the female mice. CYP11A1 and StAR, which metabolise cholesterol to pregnenolone, were unchanged at both mRNA and protein levels. Then, the SRD5A1, 5α-DHP, and allopregnanolone, which are the downstream components of the neurosteroid biosynthesis pathway, were increased. These results indicate that pregnenolone production remained unaffected, while pregnenolone consumption was increased under neuroinflammatory conditions. Evidence suggests that pregnenolone exhibits neuroprotective properties in various CNS disorders, including depression, schizophrenia, cognitive impairment, and anxiety [44, 52, 53, 55, 56]. In our study, the administration of exogenous pregnenolone effectively alleviated depressive-like behaviors and hypothalamic neuroinflammation in female mice with depression. In addition, we observed SRD5A1 upregulation in the hypothalamus of female mice with depression after pregnenolone administration. Likewise, inhibition of SRD5A1, which decreases pregnenolone consumption, resulted in elevated levels of hypothalamic pregnenolone and improved depressive-like behaviors in female mice with depression. It is plausible that the mechanism underlying this neuroprotection involves anti-inflammatory effects, promotion of neuroregeneration, and modulation of excitatory and inhibitory neurotransmission mediated by ligand-gated ion channels, such as gamma-aminobutyric acid type A, N-methyl-D-aspartate, and glucocorticoid receptors [53, 54, 56, 57]. Overall, maintaining homeostasis of the neurosteroid biosynthesis pathway is crucial for the hypothalamus in female mice. In addition, the clinical implications and potential side effects of inhibiting the 5α-reductase [38, 58], including SRD5A1, SRD5A2, and SRD5A3, have attracted considerable interest in neuropsychiatric disorders [47, 59, 60]. SRD5A1 is constitutively expressed in both peripheral and brain tissues and is predominantly localized within the myelin membranes. Its main function is to regulate the equilibrium between the conversion and production of neurosteroids within the CNS, specifically the degradation of pregnenolone and progesterone, and the synthesis of allopregnanolone [38]. Our study suggests that inhibition of SRD5A1 results in an increase in hypothalamic pregnenolone levels and a decrease in allopregnanolone levels. Pregnenolone has neuroprotective effects against cannabinoids [43] and mitigates acute psychotic-like states in mice induced by cannabinoids [61]. Decreased pregnenolone levels in the hypothalamus have been linked to cognitive decline [44] and the present study links pregnenolone to depressive-like behaviors. A reduction in allopregnanolone levels leads to heightened anxiety, increased susceptibility to seizures, and diminished myelination. 5α-reductase plays a crucial role in body development, and is also involved in pregnancy, childbirth, and glucocorticoid clearance [38, 58]. Consequently, the safety concerns associated with inhibiting the SRD5A1, as well as the potential of using such inhibitors as antidepressants, necessitate further investigation.

Hypothalamic functions are closely connected to estrous cyclicity [62], a significant variable to consider when examining sex differences in depression that are influenced by the hypothalamus. During the initiation of inflammation, the secretion of hormones associated with the estrous cycle, such as gonadotropin-releasing hormone and luteinizing hormone, is suppressed [63]. The impact of these changes in the estrous cycle on the metabolic status of the hypothalamus, particularly of lipid metabolism, and whether it is a direct or indirect effect, remains uncertain. Therefore, further investigation is crucial to elucidate alterations in the metabolic status of the hypothalamus during depression induced by inflammation or other stressors in various stages of life, such as prepuberty, puberty, pre- and post-partum periods, and the menopause. These hypothalamic changes may underlie the sex disparities observed in depression.

This study has several limitations that should be acknowledged. First, although the observed variations in hypothalamic neurosteroid metabolites were found to correlate with behavioral phenotypes in mice, it is crucial to substantiate these findings through metabolomics analysis of plasma, cerebrospinal fluid, or *post-mortem* hypothalamic tissues collected from both female and male patients diagnosed with depression. Second, it is yet to be determined whether neuroactive steroids can function as diagnostic biomarkers for the detection of depression in women. Third, we observed a significant increase in the expression of SRD5A1, a crucial enzyme in the neurosteroid pathway, in mice subjected to inflammatory stimuli. These findings were derived from the use of wild-type mice. To gain a more comprehensive understanding of the precise cellular mechanisms underlying sex differences, future investigations should also examine *Srd5a1* knockout mice. Furthermore, well-established animal models of depression, such as the chronic mild stress model, the chronic social defeat stress model, and the LPS model of depression, have been extensively employed to study the mechanisms underlying depression and to screen for antidepressants [64, 65]. Notably, the LPS-induced depressive-like model specifically mimics inflammation-associated depression, which is the predominant phenotype observed in patients with depression [5, 6]. However, it remains unclear whether the dysregulation of the steroidogenic pathway in the hypothalamus of female mice is limited to neuroinflammatory infiltration or extends to other depression models. Elucidating this aspect may contribute to the understanding of sex-specific variations in the development of antidepressants or the treatment of depression [25]. Future studies should address this using multiple animal models of depression.

## Conclusions

We identified a marked sexual dimorphism of metabolic signatures, particularly hypothalamic neurosteroidogenic metabolism, in depression as a potential sex-specific pathway in female mice with depressive-like behavior. This study provides a framework to investigate the pathological mechanisms of depression in women with and highlights the importance of studying sex-specific treatments for depression.

### Supplementary Information

**Additional file 1: Table S1.** Primer pairs for qRT-PCR.

**Additional file 2: Table S2.** Statistical parameters, including sample size (n), t/F value, p or adjusted p value, and the analysis method used for each experiment, related to Figures 1, 5, 6, 7 and S5–S6.

**Additional file 3:** Fig. S1. Hypothalamic metabolomics analysis of male and female mice, utilizing both detection modes. Fig. S2. Identified differential metabolites (DMs) in the hypothalamus from male and female mice. Fig. S3. Phosphatidylethanolamine (PE) metabolites alterations in the hypothalamus of male and female mice with LPS-evoked depression. Fig. S4. Comparative analyses of differential metabolites (DMs) in biological processes and pathways using MetaboAnalyst 5.0. Fig. S5. Quantitation of the hypothalamic neurosteroids pregnenolone, progesterone, and allopregnanolone and correlation analysis with behaviors. Fig. S6. Effects of central pregnenolone infusion (A–C) or inhibition of 5α-reductase (D–G) on hypothalamic inflammation and depressive-like behaviors in male mice.

**Additional file 4: Table S3–S6.** Significantly differential metabolites are responsible for the discrimination between different comparison group. **Table S3**. CON-Female group and CON-Male group; **Table S4**. LPS-Male group and CON-Male group; **Table S5**. LPS-Female group and CON-Female group; and **Table S6**. LPS-Female group and LPS-Male group.

**Additional file 5. **Original western blot image

## Data Availability

All data are available in the main text and supplementary materials. The original western blot images have been deposited as Additional file 5. This paper does not report original code. Any additional information required to reanalyze the data reported in this work is available from the corresponding author (Y.W.) upon request.

## Abbreviations

5α-DHP: 5Alpha-pregnane-3,20-dione
ANOVA: Analysis of variance
BP: Biological process
BW: Body weight
CNS: Central nervous system
CRP: C-reactive protein
CYP: Cytochrome P450
DM: Differential metabolite
ELISA: Enzyme-linked immunosorbent assay
ESI: Electrospray ionisation
FA: Fatty acyl
GP: Glycerophospholipid
HPA: Hypothalamic–pituitary–adrenal
HRMS/MS: High-resolution tandem mass spectrometry
i.c.v.: Intracerebroventricular
i.p.: Intraperitoneal
IL: Interleukin
LPA: Lysophosphatidic acid
LPC: Lysophosphatidylcholine
LPE: Lysophosphatidylethanolamine
LPI: Lysophosphatidylinositol
LPS: Lipopolysaccharide
LyPS: Lysophosphatidylserine
MDD: Major depressive disorder
OPLS-DA: Orthogonal partial least-squares discriminant analysis
PA: Phosphatidic acid
PBS: Phosphate-buffered saline
PC: Phosphatidylcholine
PE: Phosphatidylethanolamine
PG: Phosphatidylglycerol
PI: Phosphatidylinositol
PS: Phosphatidylserine
qRT-PCR: Quantitative real-time PCR
SEM: Standard error of the mean
SP: Sphingolipid
SPT: Sucrose preference test
ST: Sterol lipid
TNF-α: Tumour necrosis factor-alpha
WB: Western blotting
UHPLC: Ultra-high-performance liquid chromatography
VIP: Variable importance in projection
