# Supplementary material for: The hypothalamic steroidogenic pathway mediates susceptibility to inflammation-evoked depression in female mice
Source: J Neuroinflammation. 2023 Dec 7;20:293. doi: 10.1186/s12974-023-02976-7 (PMC10704691; doi:10.1186/s12974-023-02976-7)
Supplement: Supplementary file 1 — Additional file 1: Table S1. Primer pairs for qRT-PCR. [file 12974_2023_2976_MOESM1_ESM.docx]

| Table S1. Primer pairs for qRT-PCR. | | | |
| --- | --- | --- | --- |
| Gene symbol | Gene names | Primer Pairs | From 5’ to 3’ |
| *Tnf* | Tumor necrosis factor-alpha | Forward | GCCTATGTCTCAGCCTCTTCTC |
|  |  | Reverse | GCCATTTGGGAACTTCTCATCC |
| *Il1b* | Interleukin-1 beta | Forward | TCTTGGGACTGATGCTGGTG |
|  |  | Reverse | CAGAATTGCCATTGCACAACTC |
| *Il6* | Interleukin-6 | Forward | GCCTTCTTGGGACTGATGCT |
|  |  | Reverse | GCCATTGCACAACTCTTTTCTC |
| *Star* | Steroidogenic acute regulatory protein | Forward | ATGTTCCTCGCTACGTTCAAG |
|  |  | Reverse | CCCAGTGCTCTCCAGTTGAG |
| *Cyp11a1* | Cytochrome P450, family 11, subfamily a, polypeptide 1 | Forward | AGGTCCTTCAATGAGATCCCTT |
|  |  | Reverse | TCCCTGTAAATGGGGCCATAC |
| *Cyp21a1* | Cytochrome P450, family 21, subfamily a, polypeptide 1 | Forward | AGACCCTTCACGACTGTGTC |
|  |  | Reverse | CCGACTCTCTTGGATCTGCTT |
| *Srd5a3* | Steroid 5 alpha-reductase 3 | Forward | CTACGTCATCTCAGTTGTGTGG |
|  |  | Reverse | GAGCAGAGCACTAAGCCAGT |
| *Srd5a1* | Steroid 5 alpha-reductase 1 | Forward | GAGTTGGATGAGTTGCGCCTA |
|  |  | Reverse | GGACCACTGCGAGGAGTAG |
| *Srd5a2* | Steroid 5 alpha-reductase 2 | Forward | GATCCTGTGCTTTGGGAAACC |
|  |  | Reverse | GCATCCCTACCGACACCAC |
| *Cyp11b1* | Cytochrome P450, family 11, subfamily b, polypeptide 1 | Forward | CAGATTGTGTTTGTGACGTTGC |
|  |  | Reverse | CGGTTGAAGTACCATTCTGGC |
| *Cyp11b2* | cytochrome P450, family 11, subfamily b, polypeptide 2 | Forward | TGGCTGAAGATGATACAGATCCT |
|  |  | Reverse | CACTGTGCCTGAAAATGGGC |
| *β-Actin* | beta-Actin | Forward | GCCACCAGTTCGCCATGGAT |
|  |  | Reverse | TCTGGGCCTCGTCACCCACATA |
