# Supplementary material for: The hypothalamic steroidogenic pathway mediates susceptibility to inflammation-evoked depression in female mice
Source: J Neuroinflammation. 2023 Dec 7;20:293. doi: 10.1186/s12974-023-02976-7 (PMC10704691; doi:10.1186/s12974-023-02976-7)
Supplement: Supplementary file 3 — Additional file 3: Fig. S1. Hypothalamic metabolomics analysis of male and female mice, utilizing both detection modes. Fig. S2. Identified differential metabolites (DMs) in the hypothalamus from male and female mice. Fig. S3. Phosphatidylethanolamine (PE) metabolites alterations in the hypothalamus of male and female mice with LPS-evoked depression. Fig. S4. Comparative analyses of differential metabolites (DMs) in biological processes and pathways using MetaboAnalyst 5.0. Fig. S5. Quantitation of the hypothalamic neurosteroids pregnenolone, progesterone, and allopregnanolone and correlation analysis with behaviors. Fig. S6. Effects of central pregnenolone infusion (A–C) or inhibition of 5α-reductase (D–G) on hypothalamic inflammation and depressive-like behaviors in male mice. [file 12974_2023_2976_MOESM3_ESM.docx]

**Additional file 3: figures**

**
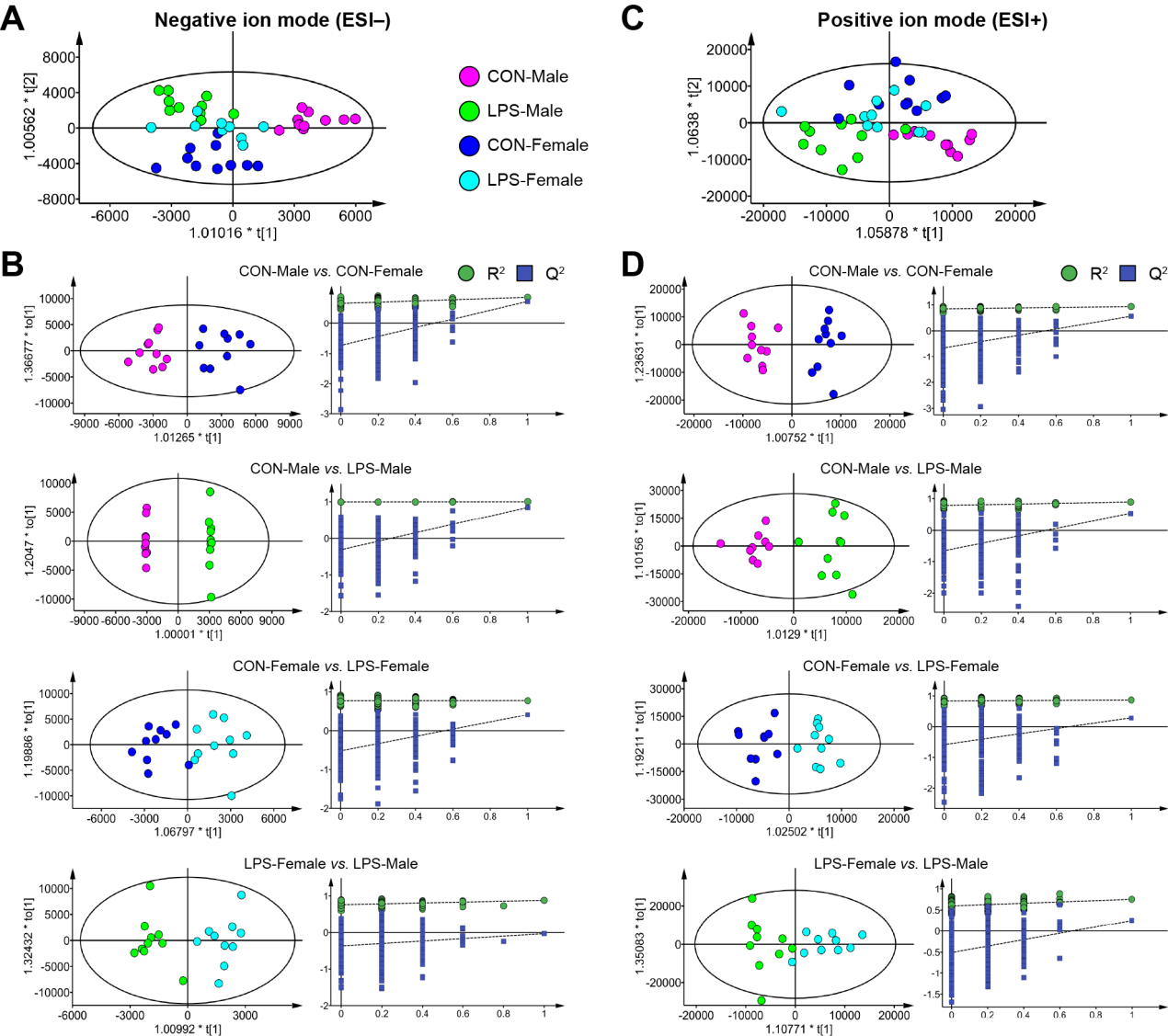
**

**Fig. S1** Hypothalamic metabolomics analysis of male and female mice, utilizing both detection modes. Related to Figure 2. (**A**, **C**) Score plot of OPLS-DA models in negative (**A**) and positive (**C**) ion modes among the four study groups, saline-treated male controls (CON-Male, purple), LPS-induced depressive-like male mice (LPS-Male, green), saline-treated female controls (CON-Female, blue), and LPS-treated depressive-like female mice (LPS-Female, turquoise). (**B**, **D**) Score plot and permutation test of OPLS-DA model in negative (**B**) and positive (**D**) ion mode in different comparisons.


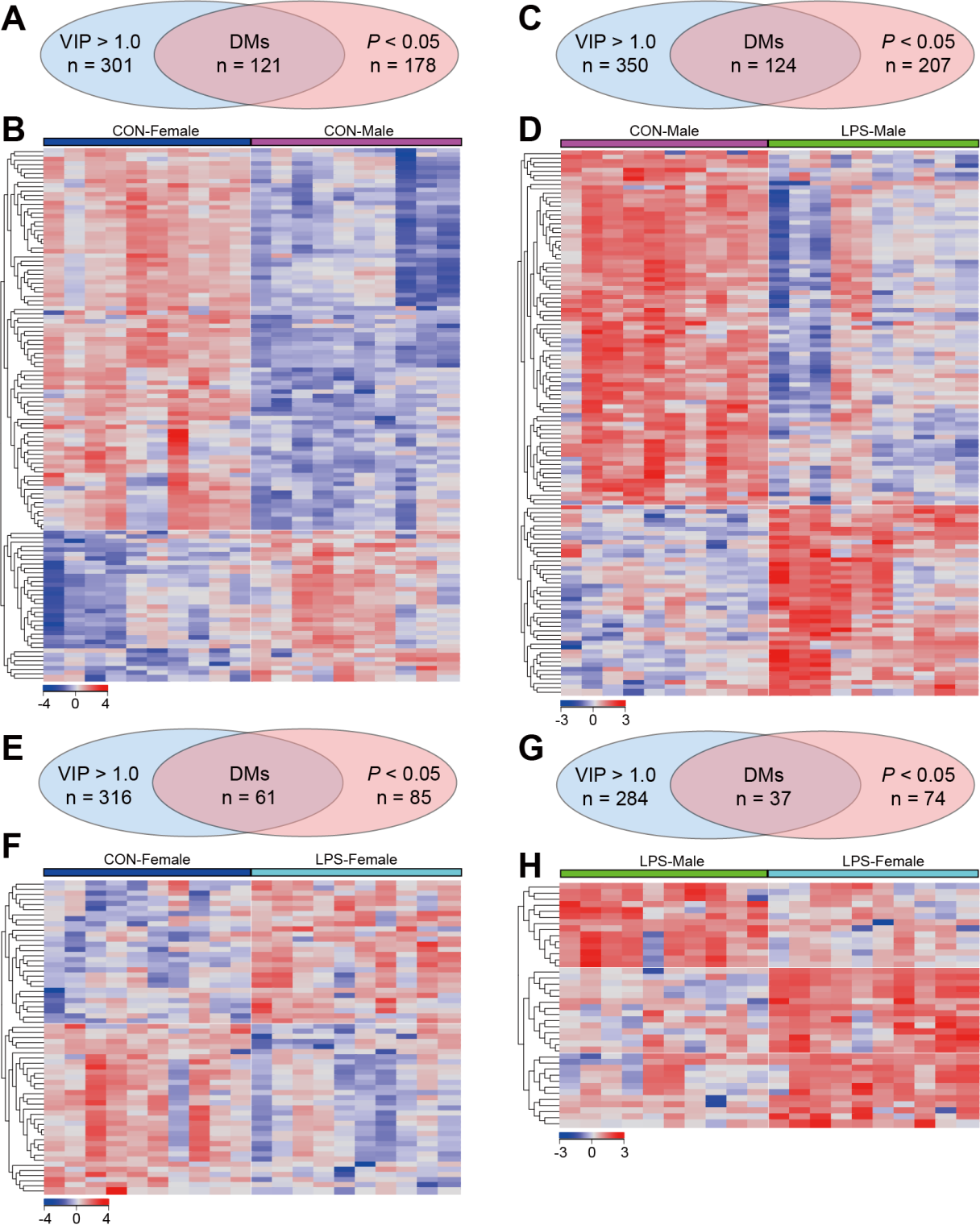


**Fig. S2** Identified differential metabolites (DMs) in the hypothalamus from male and female mice. Related to Figure 3. (A–H) Venn diagrams revealed the number of DMs (VIP > 1.0 and *P* < 0.05), and heatmaps visualize a sharp distinction between each of the two groups, including CON-Female *vs.* CON-Male (**A**, **B**), LPS-Male *vs.* CON-Male (**C**, **D**), LPS-Female *vs.* CON-Female (**E**, **F**), and LPS-Female *vs.* LPS-Male (**G**, **H**). VIP, variable importance in the projection.

**
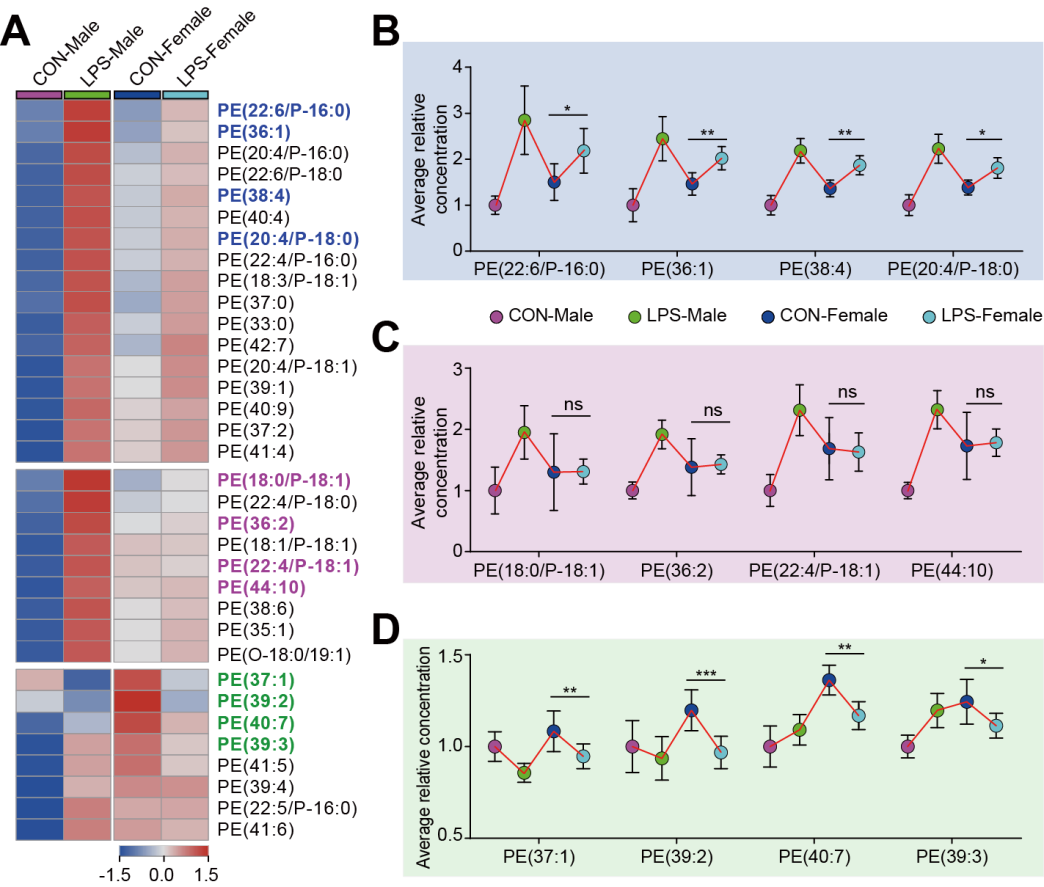
**

Fig. S3 Phosphatidylethanolamine (PE) metabolites alterations in the hypothalamus of male and female mice with LPS-evoked depression. Related to Figures 3 and 4. (A) Heatmap of differential PE lipids in the hypothalamus of LPS-induced depressive-like mice and sex-matched controls. (B–D) Distinct expression patterns of differential PE isoforms exhibiting a significant increase (B), no changed (C), or a significant decrease (D) in the hypothalamus of female mice under neuroinflammatory conditions.

**
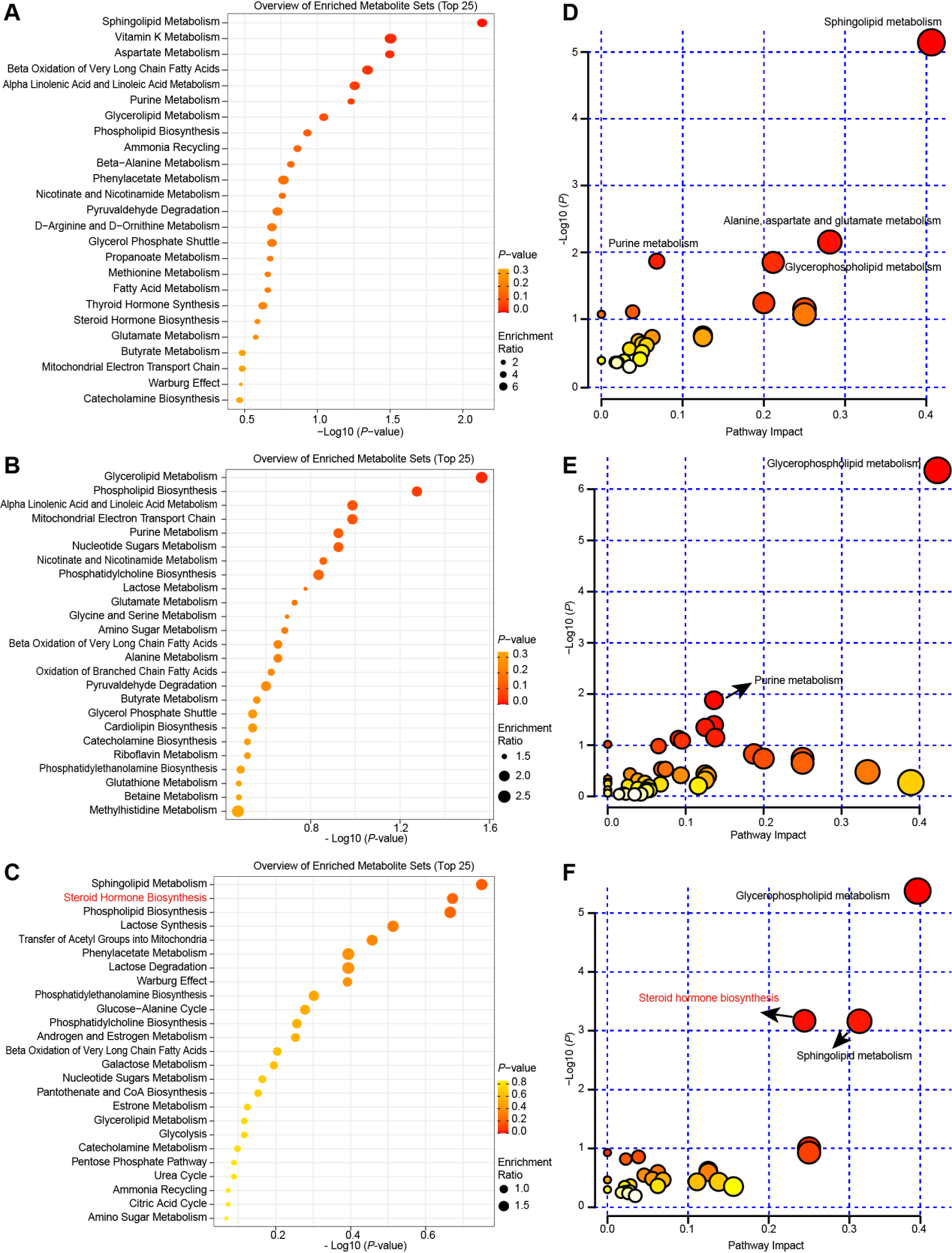
**

Fig. S4 Comparative analyses of differential metabolites (DMs) in biological processes and pathways using MetaboAnalyst 5.0**.** Related to Figure 4. (**A–C**) Metabolite set enrichment analysis and (**D–F**) metabolic pathway analysis in different comparisons, including CON-Female *vs.* CON-Male (**A**, **D**), LPS-Male *vs.* CON-Male (**B**, **E**), LPS-Female *vs.* CON-Female (**C**, **F**).


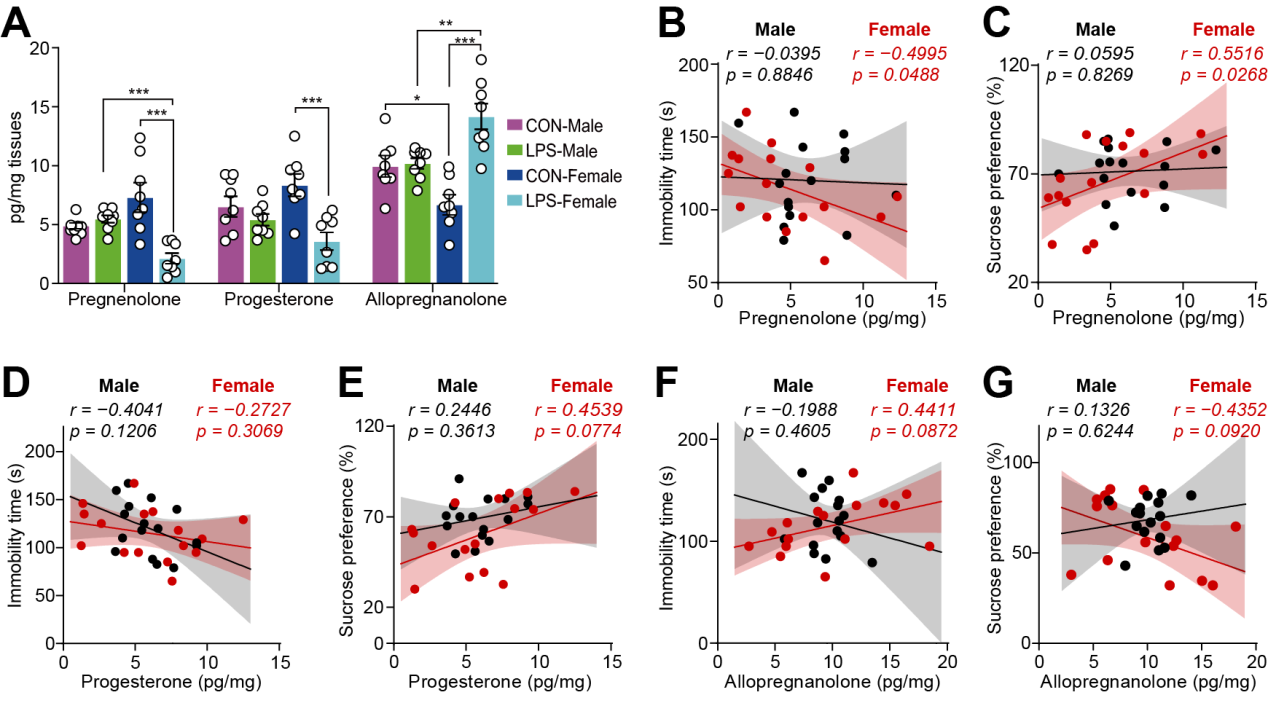


Fig. S5 Quantitation of the hypothalamic neurosteroids pregnenolone, progesterone, and allopregnanolone and correlation analysis with behaviors. Related to Figure 5. (**A**) Quantitation of hypothalamic pregnenolone, progesterone, and allopregnanolone, n = 8 mice/group. (**B–G**) Correlation analysis between these three neurosteroids and behaviors, including sucrose preference in the SPT and immobility time in the FST, n = 16 males and 16 females; Dots in panels represent individual samples. Data are presented as means ± SEM and were analyzed using two-way ANOVA (sex × treatment) followed by Bonferroni's post hoc tests (**A**) and Pearson’s correlation coefficient (*r*) and probability (*p*) tests (**B–G**). **P* < 0.05, ***P* < 0.01, ****P* < 0.001.


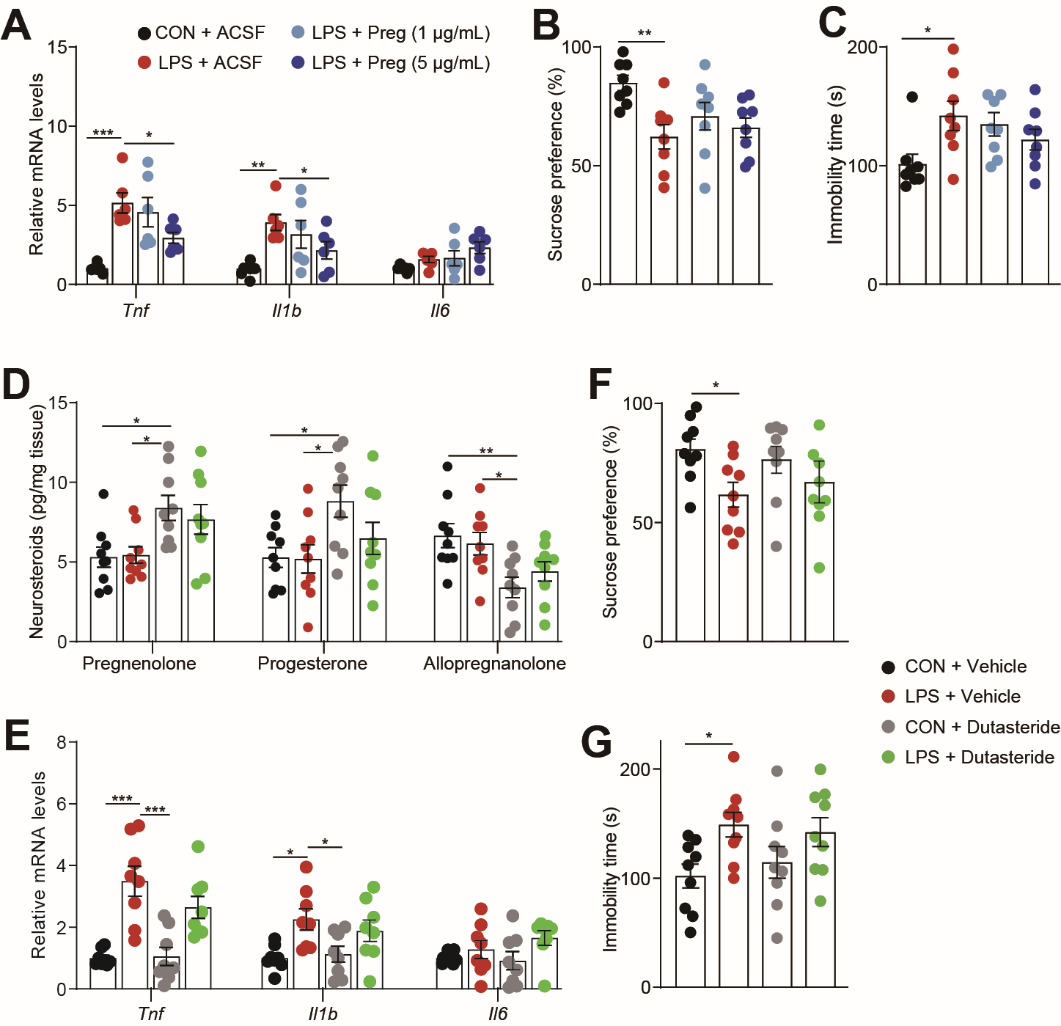


Fig. S6 Effects of central pregnenolone infusion (A–C) or inhibition of 5α-reductase (D–G) on hypothalamic inflammation and depressive-like behaviors in male mice. Related to Figures 6 and 7. (**A**) The transcriptional levels of hypothalamic *Tnf*, *Il1b*, and *Il6*, after intracerebroventricular (i.c.v.) administration of artificial cerebrospinal fluid (ACSF) or pregnenolone (Preg), n = 6 mice/group. (**B**, **C**) Recorded parameters to assess sucrose preference (**B**) in the SPT and immobility (**C**) in the FST after infusion of ACSF or pregnenolone, n = 8 mice/group. (**D**) Hypothalamic levels of the three neurosteroids, pregnenolone, progesterone, and allopregnanolone, in male mice after infusion of vehicle or dutasteride, n = 9 mice/group. (**E**) Transcriptional levels of hypothalamic *Tnf*, *Il1b*, and *Il6* expression, n = 8 mice/group. (**F, G**) Behavioral assessments: sucrose preference (**F**) and immobility (**G**), n = 9 mice/group. Data are presented as means ± SEM and were analyzed using one-way ANOVA. **P* < 0.05, ***P* < 0.01, ****P* < 0.001.
