# Supplementary material for: The hypothalamic steroidogenic pathway mediates susceptibility to inflammation-evoked depression in female mice
Source: J Neuroinflammation. 2023 Dec 7;20:293. doi: 10.1186/s12974-023-02976-7 (PMC10704691; doi:10.1186/s12974-023-02976-7)
Supplement: Supplementary file 4 — Additional file 4: Table S3–S6. Significantly differential metabolites are responsible for the discrimination between different comparison group. Table S3. CON-Female group and CON-Male group; Table S4. LPS-Male group and CON-Male group; Table S5. LPS-Female group and CON-Female group; and Table S6. LPS-Female group and LPS-Male group. [file 12974_2023_2976_MOESM4_ESM.pdf]

Table S3. Significantly differential metabolites are responsible for the discrimination between CON-Female group and CON-Male group.

Class	Name	Compound ID	VIP	RT (min)	m/z	Ion mode	Formula	*P*	Log_2FC
Carboxylic acids and derivatives	D-Pipecolic acid	HMDB0005960	2.09	0.59	147.11	POS	C6H11NO2	7.534E-04	0.281
	N-Acetylaspartylglutamic acid	HMDB0001067	7.55	1.12	303.08	NEG	C11H16N2O8	2.127E-02	-0.292
	L-Glutamine	HMDB0000641	1.90	0.65	169.06	POS	C5H10N2O3	5.910E-05	-0.351
	Methylmalonic acid	HMDB0000202	2.13	0.66	163.02	NEG	C4H6O4	7.648E-03	-0.401
	N2-Succinyl-L-ornithine	HMDB0001199	1.13	1.09	233.11	POS	C9H16N2O5	7.443E-03	-0.411
	N-Acetyl-L-aspartic acid	HMDB0000812	1.66	0.91	349.09	NEG	C6H9NO5	8.249E-03	-0.417
	Garcinia acid	HMDB0031159	2.41	0.68	207.01	NEG	C6H8O8	1.173E-02	-0.446
	Pipecolic acid	HMDB0000070	1.57	0.97	130.09	POS	C6H11NO2	5.655E-04	-1.111
	Methionine sulfoxide	HMDB0002005	1.53	1.28	329.09	NEG	C5H11NO3S	1.336E-02	-1.126
Fatty Acyls	Tetracosanoic acid	HMDB0002003	2.52	10.35	367.36	NEG	C24H48O2	1.099E-07	1.662
	Docosanoic acid	HMDB0000944	4.68	8.16	358.37	POS	C22H44O2	3.385E-05	1.619
	24-hydroxy-tetracosanoic acid	LMFA01050213	3.55	8.18	402.39	POS	C24H48O3	8.059E-05	1.181
	Nervonic acid	HMDB0002368	1.07	9.84	365.34	NEG	C24H46O2	1.636E-03	0.735
	Palmitoleic acid	HMDB0003229	2.10	4.38	272.26	POS	C16H30O2	1.175E-02	0.315
	Vaccenyl carnitine	HMDB0006351	4.89	7.20	426.36	POS	C25H47NO4	1.988E-02	-0.313
	Palmitoylcarnitine	HMDB0000222	6.25	6.90	400.34	POS	C23H45NO4	5.367E-03	-0.371
	Dihomo-γ-Linolenic acid	HMDB0002925	1.11	8.06	305.25	NEG	C20H34O2	8.553E-03	-0.381
	Docosapentaenoic acid (22n-6)	HMDB0001976	1.25	8.01	329.25	NEG	C22H34O2	5.736E-03	-0.431
	Acetylcarnitine	HMDB0000201	3.80	1.00	204.12	POS	C9H17NO4	1.377E-02	-0.627
	Dodecanoylcarnitine	HMDB0002250	1.89	4.61	344.28	POS	C19H37NO4	6.590E-04	-0.931
	Butyrylcarnitine	HMDB0002013	1.26	2.35	232.15	POS	C11H21NO4	2.955E-04	-1.033
	cis-5-Tetradecenoylcarnitine	HMDB0002014	2.11	4.99	370.30	POS	C21H39NO4	6.994E-04	-1.077
	Tetradecanoylcarnitine	HMDB0005066	4.13	5.63	372.31	POS	C21H41NO4	8.071E-03	-0.629
	Tetradecanoylcarnitine	HMDB0005066	4.47	5.63	372.31	POS	C21H42NO4	8.071E-03	-0.629
	trans-Hexadec-2-enoyl carnitine	HMDB0006317	3.70	5.90	398.33	POS	C23H43NO4	2.093E-04	-0.788
	24-hydroxy-10Z-tetracosenoic acid	LMFA01050215	1.30	6.69	400.38	POS	C24H46O3	9.672E-06	-0.876

Flavin nucleotides	Flavin adenine dinucleotide (FAD)	HMDB0001248	1.20	2.41	784.15	NEG	C27H33N9O15	1.663E-02	-0.319
Glycerophospholipids	PE(22:6/P-16:0)	HMDB0009708	10.67	10.08	746.51	NEG	C43H74NO7P	3.354E-02	1.682
	PE(42:7)	HMDB0009276	1.58	12.24	840.55	POS	C47H80NO8P	1.663E-03	1.540
	PG(29:0)	LMGP04010050	2.99	10.79	698.50	POS	C35H69O10P	1.685E-02	1.309
	PG(31:0)	LMGP04010076	2.24	10.79	726.53	POS	C37H73O10P	3.499E-02	1.296
	PE(33:0)	HMDB0008892	3.55	10.04	750.53	NEG	C38H76NO8P	6.490E-05	1.243
	PE(40:4)	HMDB0009009	3.74	10.81	794.57	NEG	C45H82NO8P	3.549E-05	1.226
	PE(44:10)	HMDB0009605	3.23	9.75	838.54	NEG	C49H78NO8P	6.320E-08	1.214
	PE(22:4/P-16:0)	HMDB0009609	4.00	10.57	750.54	NEG	C43H78NO7P	2.029E-06	1.213
	PC(42:9)	HMDB0008387	3.01	11.66	878.57	POS	C50H82NO8P	6.779E-03	1.211
	PE(22:4/P-18:1)	HMDB0009611	3.53	10.56	776.56	NEG	C45H80NO7P	9.689E-06	1.209
	PE(38:6)	HMDB0008946	9.00	9.91	762.51	NEG	C43H74NO8P	5.275E-06	1.196
	PE(36:1)	HMDB0008992	4.88	11.07	744.56	NEG	C41H80NO8P	1.743E-04	1.168
	PE(40:4)	HMDB0009322	2.09	11.07	812.54	NEG	C43H78NO8P	1.926E-04	1.116
	LPE(20:4)	HMDB0011517	3.49	6.67	500.28	NEG	C25H44NO7P	1.565E-06	1.102
	PE(40:7)	HMDB0009685	4.14	9.91	788.52	NEG	C45H76NO8P	2.539E-07	1.072
	PnE(34:1)	LMGP17010001	4.93	10.81	700.53	NEG	C39H76NO7P	2.000E-04	1.062
	PE(20:4/P-16:0)	HMDB0009411	3.59	10.27	722.51	NEG	C41H74NO7P	4.817E-05	1.043
	PE(22:4/P-18:0)	HMDB0009610	3.81	11.03	778.58	NEG	C45H82NO7P	1.191E-04	1.020
	PE(20:4/P-18:0)	HMDB0009412	5.38	10.70	750.54	NEG	C43H78NO7P	1.170E-05	1.019
	PI(42:3)	LMGP06010523	1.48	9.23	962.67	POS	C51H93O13P	3.172E-04	1.008
	PE(38:4)	HMDB0009003	6.77	10.50	766.54	NEG	C43H78NO8P	4.090E-06	0.987
	PG(27:0)	LMGP04010047	3.20	10.01	670.47	POS	C33H65O10P	1.915E-02	0.987
	PE(20:4/P-18:1)	HMDB0009413	6.19	10.25	748.53	NEG	C43H76NO7P	1.423E-06	0.964
	PE(18:0/P-18:1)	HMDB0009017	4.18	11.32	728.56	NEG	C41H80NO7P	1.596E-03	0.964
	PE(22:6/P-18:0)	HMDB0009709	6.66	10.51	774.54	NEG	C45H78NO7P	2.736E-05	0.954
	PE(36:2)	HMDB0009025	3.96	10.57	742.54	NEG	C41H78NO8P	4.771E-07	0.939
	PE(O-18:0/19:1)	LMGP02020053	2.95	10.76	790.60	NEG	C42H84NO7P	2.866E-05	0.908

Glycerophospholipids	PE(18:1/P-18:1)	HMDB0009050	4.55	10.79	726.55	NEG	C41H78NO7P	9.749E-05	0.854
	PE(37:0)	LMGP02010067	6.54	10.96	806.59	NEG	C42H84NO8P	3.625E-04	0.852
	PE(18:3/P-18:1)	HMDB0009149	1.80	10.81	768.52	NEG	C41H74NO7P	2.810E-04	0.782
	LPE(18:0)	HMDB0011130	2.51	7.86	480.31	NEG	C23H48NO7P	1.133E-06	0.778
	PE(40:9)	HMDB0009144	3.12	9.91	830.50	NEG	C45H72NO8P	1.006E-07	0.748
	LyPS(16:0)	LMGP03050002	1.61	5.38	498.28	POS	C22H44NO9P	6.104E-05	0.633
	PC(40:6)	HMDB0008057	4.65	10.23	878.59	NEG	C48H84NO8P	1.609E-08	0.627
	PC(42:10)	HMDB0008452	3.45	12.22	854.57	POS	C50H80NO8P	2.152E-02	0.585
	PE(22:5/P-16:0)	HMDB0009642	1.24	10.79	794.53	NEG	C43H76NO7P	6.120E-04	0.581
	PI(40:3)	LMGP06010331	1.83	9.23	934.64	POS	C49H89O13P	2.412E-02	0.553
	LPE(O-18:1)	LMGP02060004	2.60	8.24	464.31	NEG	C23H48NO6P	1.753E-05	0.538
	PC(33:0)	HMDB0007937	2.22	11.67	770.57	POS	C41H82NO8P	7.177E-03	0.469
	PE(41:4)	LMGP02010791	4.84	10.38	854.59	NEG	C46H84NO8P	7.732E-06	0.469
	LPI(18:0)	HMDB0240262	3.81	6.09	601.33	POS	C27H53O12P	1.558E-06	0.468
	LyPS(22:4)	LMGP03050014	2.94	5.68	574.31	POS	C28H48NO9P	1.331E-06	0.460
	LPC(P-15:0)	LMGP01070003	1.09	6.05	466.33	POS	C23H48NO6P	5.534E-03	0.449
	PS(39:2)	LMGP03010246	1.70	10.97	874.58	NEG	C45H84NO10P	2.443E-02	0.439
	PC(32:0)	LMGP01010537	5.04	10.50	778.56	NEG	C40H80NO8P	2.928E-03	0.434
	LPE(16:0)	HMDB0011503	6.00	5.56	454.29	POS	C21H44NO7P	7.169E-07	0.422
	LPC(20:4)	HMDB0010395	1.35	6.59	588.33	NEG	C28H50NO7P	1.854E-02	0.410
	PE(35:1)	LMGP02010571	4.11	10.05	776.55	NEG	C40H78NO8P	4.559E-04	0.396
	PE(39:1)	LMGP02010812	5.19	10.93	832.61	NEG	C44H86NO8P	8.621E-05	0.393
	LyPS(20:4)	LMGP03050007	2.02	4.98	546.28	POS	C26H44NO9P	2.036E-04	0.378
	PC(O-16:1/2:0)	LMGP01020147	7.39	5.65	522.36	POS	C26H52NO7P	1.234E-02	0.359
	PE(41:6)	LMGP02010792	4.89	9.82	850.56	NEG	C46H80NO8P	7.945E-06	0.338
	LPC(18:1)	HMDB0002815	2.28	7.19	566.35	NEG	C26H52NO7P	3.003E-02	0.337
	LPE(16:1)	HMDB0011504	1.18	4.88	452.28	POS	C21H42NO7P	1.138E-02	0.287
	LPC(22:4)	HMDB0010401	2.58	5.88	572.37	POS	C30H54NO7P	2.219E-02	0.285

Glycerophospholipids	PC(40:7)	HMDB0008090	1.97	9.82	876.58	NEG	C48H82NO8P	3.179E-04	0.272
	PE(41:5)	LMGP02010822	1.50	9.97	852.58	NEG	C46H82NO8P	8.937E-04	0.259
	PC(30:1)	HMDB0007870	1.36	8.16	704.52	POS	C38H74NO8P	8.080E-03	-0.405
	PC(38:6)	HMDB0007991	32.37	9.44	806.57	POS	C46H80NO8P	1.247E-02	-0.428
	PA(O-20:0/17:2)	LMGP10020051	1.20	12.22	718.57	POS	C40H77O7P	1.527E-02	-0.676
	PS(37:3)	LMGP03010189	1.23	10.04	844.53	NEG	C43H78NO10P	3.264E-02	-0.714
	PC(38:2)	HMDB0008077	3.26	9.82	814.63	POS	C46H88NO8P	4.120E-02	-0.904
	PS(39:3)	LMGP03010275	2.45	10.44	872.56	NEG	C45H82NO10P	2.542E-04	-0.910
	PS(39:4)	LMGP03010247	1.16	10.11	870.55	NEG	C45H80NO10P	1.059E-03	-1.113
Sterol Lipids	3-Deoxyvitamin D3	LMST03020618	12.58	11.66	369.35	POS	C27H44	6.295E-04	2.032
	5alpha-pregnane-3,20-dione	LMST02030170	1.04	4.44	317.25	POS	C21H32O2	6.785E-03	0.989
	Minabeolide-7	LMST01160008	1.30	10.64	911.57	NEG	C28H40O5	3.356E-05	-0.901
Sphingolipids	Cer(d16:1/17:0)	LMSP02010015	1.42	11.67	524.50	POS	C33H65NO3	1.370E-04	3.747
	Cer(d18:1/14:0)	HMDB0011773	1.16	11.12	510.49	POS	C32H63NO3	1.385E-04	2.205
	Cer(d18:1/18:0)	HMDB0004950	16.35	10.90	610.54	NEG	C36H71NO3	2.337E-05	2.047
	Cer(d18:1/18:1)	HMDB0004948	9.11	10.40	608.53	NEG	C36H69NO3	4.725E-05	1.786
	GlcCer(d18:0/16:0)	LMSP0501AA04	1.14	10.73	724.57	POS	C40H79NO8	2.390E-03	1.654
	GlcCer(d14:2/22:0)	LMSP0501AA56	3.03	10.98	726.59	POS	C42H79NO8	1.333E-03	1.597
	Sphinganine	HMDB0000269	2.03	5.86	302.31	POS	C18H39NO2	2.938E-10	1.589
	2-amino-14,16-dimethyloctadecan-3-ol	LMSP01080031	1.43	8.27	314.34	POS	C20H43NO	1.166E-03	1.458
	GlcCer(d14:1/22:1)	LMSP0501AA67	2.88	10.73	742.58	POS	C42H79NO9	3.172E-03	1.422
	Cer(d18:2/16:0)	LMSP02010024	2.62	11.15	536.50	POS	C34H65NO3	1.270E-03	1.389
	SM(d17:1/17:0)	LMSP03010043	3.76	10.14	747.57	NEG	C39H79N2O6P	2.844E-05	1.311
	GlcCer(d14:1/20:0)	LMSP0501AA42	3.39	10.95	700.57	POS	C40H77NO8	3.342E-03	1.283
	Sphingosine	HMDB0000252	4.74	5.53	300.29	POS	C18H37NO2	2.207E-08	1.239
	GlcCer(d18:1/18:0)	HMDB0004972	13.79	12.11	728.60	POS	C42H81NO8	2.534E-04	1.157
	GlcCer(d18:1/18:1)	LMSP0501AA27	4.27	11.82	726.59	POS	C42H79NO8	8.371E-04	1.059
	SM(d18:1/18:0)	HMDB0001348	10.29	10.61	775.60	NEG	C41H83N2O6P	1.641E-05	0.858
	GlcCer(d18:1/24:1)	HMDB0004975	4.76	11.49	854.67	NEG	C48H91NO8	2.925E-02	0.753
	SM(d18:1/18:1)	HMDB0012101	3.10	10.14	773.58	NEG	C41H81N2O6P	9.551E-04	0.577

Imidazopyrimidines	5-Hydroxyisourate	HMDB0030097	2.66	0.65	185.03	POS	C5H4N4O4	3.230E-03	-0.255
	8-Hydroxyguanine	HMDB0002032	1.17	0.66	166.04	NEG	C5H5N5O2	4.376E-03	-0.274
	Xanthine	HMDB0000292	2.88	0.99	151.03	NEG	C5H4N4O2	6.615E-03	-0.356
	Guanine	HMDB0000132	1.51	1.30	152.06	POS	C5H5N5O	1.925E-03	-0.437
Lactones	Dehydroascorbic acid	HMDB0001264	1.09	0.71	219.01	NEG	C6H6O6	1.951E-03	0.729
Organic oxoanionic compounds	5-Diphosphomevalonate	HMDB0001090	1.45	0.68	353.00	NEG	C6H14O10P2	1.655E-02	-0.495
Organonitrogen compounds	Stearoylethanolamide	HMDB0013078	1.04	7.87	328.32	POS	C20H41NO2	1.441E-03	0.375
Organooxygen compounds	Pantothenic Acid	HMDB0000210	3.87	2.30	220.12	POS	C9H17NO5	8.436E-04	0.457
	Glyceric acid	HMDB0000139	1.50	0.70	105.02	NEG	C3H6O4	6.756E-03	-0.723

LPC, lysophosphatidylcholine; LPE, lysophosphatidylethanolamine; LPI, lysophosphatidylinositol; LyPS, lysophosphatidylserine; PA, phosphatidic acid; PC, phosphatidylcholine; PE, phosphatidylethanolamine; PG, phosphatidylglycerol; PI, phosphatidylinositol; PS, phosphatidylserine; VIP, variable importance in projection; FC, Fold change; RT, Retention time; Compound ID was mainly exhibited based on the Human Metabolome Database (HMDB, http://www.hmdb.ca) and LIPID MAPS (http://www.lipidmaps.org).

Table S4. Significantly differential metabolites responsible for the discrimination of two groups (LPS-M *vs*. CON-M).

Class	Name	Compound ID	VIP	RT (min)	m/z	Ion mode	Formula	*P*	Log_2FC
Sterol Lipids	16alpha-Hydroxy DHEA 3-sulfate	HMDB0000386	1.13	7.12	385.17	POS	C19H28O6S	1.593E-03	-0.317
	7-Sulfocholic acid	HMDB0002421	1.04	5.65	511.23	POS	C24H40O8S	1.046E-03	-0.933
	5alpha-pregnane-3,20-dione	HMDB0003759	4.25	4.44	317.25	POS	C21H32O2	9.175E-03	-0.973
	Tetrahydrocortisone	HMDB0000903	2.16	3.88	382.26	POS	C21H32O5	2.912E-02	-1.635
	2-Methoxyestradiol	HMDB0000405	1.42	3.45	320.22	POS	C19H26O3	4.330E-02	-1.227
Sphingolipids	SM(d17:1/17:0)	LMSP03010043	1.83	10.14	747.57	NEG	C39H79N2O6P	3.143E-02	0.596
	Hexadecasphinganine	LMSP01040001	8.29	4.22	274.27	POS	C16H35NO2	8.461E-03	0.297
Pyrimidine nucleotides	Uridine	HMDB0000290	3.03	0.68	606.07	NEG	C17H27N3O17P2	1.965E-02	-0.284
	Uridine diphosphategalactose	HMDB0000302	3.49	0.68	565.05	NEG	C15H24N2O17P2	2.416E-03	-0.378
	Uridine	HMDB0000296	5.00	1.08	289.07	NEG	C9H12N2O6	8.040E-05	-0.490
	Cytidine	HMDB0000089	1.41	0.96	244.09	POS	C9H13N3O5	2.790E-05	-0.758
Pyridines and derivatives	Niacinamide	HMDB0001406	11.63	0.99	123.06	POS	C6H6N2O	6.636E-05	-0.391
	Hydroxycotinine	HMDB0001390	1.32	1.23	385.18	POS	C10H12N2O2	1.014E-03	-0.959
Purine nucleotides	Adenosine diphosphate (ADP)	HMDB0001341	1.17	0.71	426.02	NEG	C10H15N5O10P2	1.309E-02	-0.522
	Inosine	HMDB0000195	9.39	1.30	267.07	NEG	C10H12N4O5	1.612E-02	-0.580
	8-Hydroxyguanosine	HMDB0002044	1.19	1.10	322.08	POS	C10H13N5O6	7.118E-03	-0.298
	Succinyladenosine	HMDB0000912	1.77	2.26	384.11	POS	C14H17N5O8	5.897E-03	-0.505
	Guanosine	HMDB0000133	2.41	1.28	282.08	NEG	C10H13N5O5	1.574E-03	-0.767
Organooxygen compounds	Fructose 6-phosphate	HMDB0000124	1.00	0.65	283.02	POS	C6H13O9P	6.877E-05	1.288
	Galactose 1-phosphate	HMDB0000645	4.32	0.64	259.02	NEG	C6H13O9P	2.376E-04	0.458
	Phosphohydroxypyruvic acid	HMDB0001024	1.44	0.57	184.99	POS	C3H5O7P	1.492E-02	0.328
	Ribose 1-phosphate	HMDB0001489	3.76	0.66	229.01	NEG	C5H11O8P	5.781E-05	-0.309
	Glyceric acid	HMDB0000139	1.93	0.70	105.02	NEG	C3H6O4	1.376E-05	-1.344

Imidazopyrimidines	Hypoxanthine	HMDB0000157	6.91	0.71	137.05	POS	C5H4N4O	2.288E-04	-0.363
	Xanthine	HMDB0000292	3.85	0.99	151.03	NEG	C5H4N4O2	1.117E-05	-0.691
	Guanine	HMDB0000132	1.92	1.30	152.06	POS	C5H5N5O	9.913E-06	-0.871
	Adenine	HMDB0000034	1.07	1.20	134.05	NEG	C5H5N5	8.529E-04	-0.888
Glycerophospholipids	PG(29:0)	LMGP04010050	2.39	10.79	698.50	POS	C35H69O10P	1.673E-03	1.204
	PG(27:0)	LMGP04010047	3.24	10.01	670.47	POS	C33H65O10P	1.826E-03	1.177
	PG(31:0)	LMGP04010076	1.61	10.79	726.53	POS	C37H73O10P	3.609E-04	0.947
	PE(44:10)	HMDB0009605	2.05	9.75	838.54	NEG	C49H78NO8P	8.968E-03	0.790
	PE(40:7)	HMDB0009685	2.70	9.91	788.52	NEG	C45H76NO8P	1.009E-02	0.702
	PE(38:6)	HMDB0008946	5.17	9.91	762.51	NEG	C43H74NO8P	1.622E-02	0.656
	PE(20:4/P-18:1)	HMDB0009413	3.87	10.25	748.53	NEG	C43H76NO7P	2.395E-02	0.624
	LyPS(16:0)	HMDB0240605	1.27	5.38	498.28	POS	C22H44NO9P	9.554E-04	0.615
	LPG(20:5)	LMGP04050030	1.10	7.23	548.30	POS	C26H43O9P	2.236E-02	0.606
	PE(O-18:0/19:1)	LMGP02020053	1.70	10.76	790.60	NEG	C42H84NO7P	1.929E-02	0.509
	PE(40:9)	HMDB0009144	1.98	9.91	830.50	NEG	C45H72NO8P	1.535E-02	0.476
	PS(38:1)	LMGP03010199	2.82	3.77	840.57	POS	C44H84NO10P	1.534E-02	0.417
	PC(40:6)	HMDB0008057	3.30	10.23	878.59	NEG	C48H84NO8P	8.512E-04	0.402
	PC(33:0)	HMDB0007937	1.76	11.67	770.57	POS	C41H82NO8P	1.315E-02	0.402
	PE(39:3)	LMGP02010784	1.69	10.17	828.58	NEG	C44H82NO8P	1.794E-03	0.390
	PE(37:2)	LMGP02010553	1.73	10.10	802.56	NEG	C42H80NO8P	2.397E-04	0.355
	PE(35:1)	LMGP02010571	3.47	10.05	776.55	NEG	C40H78NO8P	3.182E-03	0.338
	PE(41:4)	LMGP02010791	3.50	10.38	854.59	NEG	C46H84NO8P	3.498E-03	0.321
	PE(41:5)	LMGP02010822	2.46	9.97	852.58	NEG	C46H82NO8P	7.628E-04	0.315
	LPC(14:0)	HMDB0010379	1.04	4.69	468.31	POS	C22H46NO7P	7.484E-03	0.314
	LPE(18:0)	HMDB0011130	1.31	7.86	480.31	NEG	C23H48NO7P	4.235E-03	0.304
	PE(41:6)	HMDB0009012	5.39	9.82	850.56	NEG	C46H80NO8P	1.903E-05	0.304
	PC(40:7)	HMDB0008090	2.27	9.82	876.58	NEG	C48H82NO8P	1.788E-05	0.299
	LPE(16:0)	HMDB0011503	4.12	5.56	454.29	POS	C21H44NO7P	2.347E-03	0.298

Glycerophospholipids	PE(39:4)	LMGP02010560	4.39	9.98	826.56	NEG	C44H80NO8P	3.677E-04	0.287
	PE(39:2)	LMGP02010813	1.54	10.44	830.59	NEG	C44H84NO8P	2.308E-02	0.260
	PE(39:1)	LMGP02010812	3.29	10.93	832.61	NEG	C44H86NO8P	3.840E-02	0.244
	LPI(18:1)	HMDB0061693	1.08	5.32	599.32	POS	C27H51O12P	5.913E-03	-0.262
	LPI(O-16:0)	LMGP06060003	1.00	5.32	581.31	POS	C25H51O11P	6.729E-03	-0.289
	LyPS(22:6)	LMGP03050013	2.63	7.63	568.27	NEG	C28H44NO9P	3.790E-03	-0.350
	LPG(22:6)	LMGP04050016	1.34	5.12	557.29	POS	C28H45O9P	4.818E-06	-0.449
	LPC(P-18:0)	HMDB0013122	1.03	7.05	508.38	POS	C26H54NO6P	6.355E-04	-0.461
	LPC(O-16:1)	LMGP01060028	1.75	5.95	480.34	POS	C24H50NO6P	1.687E-03	-0.462
	LPG(18:1)	HMDB0240602	1.77	5.86	511.30	POS	C24H47O9P	1.954E-04	-0.488
	LPG(O-16:0)	LMGP04060003	1.17	5.86	493.29	POS	C22H47O8P	1.289E-04	-0.519
	PA(P-16:0/16:1)	LMGP10030008	1.17	7.76	629.46	NEG	C35H67O7P	2.698E-03	-0.541
	LPG(20:4)	LMGP04050010	1.17	5.18	533.29	POS	C26H45O9P	5.836E-06	-0.579
	LPC(P-18:1)	HMDB0010408	1.13	6.09	506.36	POS	C26H52NO6P	2.565E-03	-0.595
	LPA(17:2)	LMGP10050022	1.06	5.63	421.23	POS	C20H37O7P	5.759E-03	-0.913
	PA(P-16:0/20:5)	LMGP10030022	1.37	7.62	677.46	NEG	C39H67O7P	3.183E-04	-0.975
	LPA(20:1)	HMDB0062305	1.96	7.56	465.30	POS	C23H45O7P	1.590E-08	-1.426
	LPA(18:0)	HMDB0007854	1.28	7.43	439.28	POS	C21H43O7P	1.446E-06	-1.822
	sn-glycerol 3-phosphate	HMDB0000126	3.03	0.64	171	NEG	C3H9O6P	1.178E-02	-1.273
	Glycerylphosphorylethanolamine	HMDB0000114	1.29	0.66	216.1	POS	C5H14NO6P	2.445E-03	0.571
Flavin nucleotides	Flavin adenine dinucleotide (FAD)	HMDB0001248	1.59	2.41	784.15	NEG	C27H33N9O15P2	1.345E-04	-0.598
Fatty Acyls	2-Methylcitric acid	HMDB0000379	1.15	0.85	224.08	POS	C7H10O7	1.817E-04	1.540
	Tetracosanoic acid	HMDB0002003	1.56	10.35	367.36	NEG	C24H48O2	5.067E-03	0.928
	Galactaric acid	HMDB0000639	1.22	0.64	209.03	NEG	C6H10O8	1.083E-02	0.387
	Palmitoleic acid	HMDB0003229	1.62	4.38	272.26	POS	C16H30O2	2.882E-02	0.325
	Itaconic acid	HMDB0002092	1.20	0.64	129.02	NEG	C5H6O4	7.865E-03	0.283
	Palmitoylethanolamide	HMDB0002100	1.66	6.82	300.29	POS	C18H37NO2	1.527E-02	-0.339
	S-Acetyldihydrolipoamide-E	HMDB0006878	1.11	2.24	250.09	POS	C10H19NO2S2	3.678E-03	-0.402

Fatty Acyls	Senecioic acid	HMDB0000509	1.24	0.57	123.04	POS	C5H8O2	3.633E-02	-0.415
	Arachidonic Acid	HMDB0001043	2.38	7.31	305.25	POS	C20H32O2	7.600E-05	-0.427
	Palmitic acid	HMDB0000220	1.77	8.29	255.23	NEG	C16H32O2	1.148E-04	-0.445
	Docosahexaenoic acid (DHA)	HMDB0002183	2.05	7.12	329.25	POS	C22H32O2	1.383E-04	-0.461
	2-aminomuconic acid	HMDB0001241	1.07	0.91	156.03	NEG	C6H7NO4	4.578E-05	-0.472
	(9S,10S)-10-hydroxy-9-(phosphonoo	HMDB0059632	2.48	7.62	395.22	NEG	C18H37O7P	6.629E-04	-0.476
	Elaidic Acid	HMDB0000573	4.07	8.34	281.25	NEG	C18H34O2	1.044E-03	-0.505
	3-Hydroxyisovalerylcarnitine	HMDB0061189	2.18	2.20	262.16	POS	C12H23NO5	3.282E-03	-0.528
	Dihomo-gamma-linolenic acid	HMDB0002925	1.37	8.06	305.25	NEG	C20H34O2	1.035E-03	-0.617
	12(R)-HETE	LMFA03060008	1.18	6.34	319.23	NEG	C20H32O3	1.932E-04	-0.846
	Docosapentaenoic acid (DPA)	HMDB0006528	2.09	8.01	329.25	NEG	C22H34O2	1.104E-05	-0.976
	L-Acetylcarnitine	HMDB0000201	4.85	1.00	204.12	POS	C9H17NO4	3.613E-04	-0.999
	Vaccenyl carnitine	HMDB0006351	6.11	7.20	426.36	POS	C25H47NO4	2.364E-02	-0.379
Pteridines and derivatives	7,8-Dihydroneopterin	HMDB0002275	1.38	1.06	256.10	POS	C9H13N5O4	8.610E-04	2.593
Phenylpropanoic acids	Hydroxyphenyllactic acid	HMDB0000755	1.07	2.44	181.05	NEG	C9H10O4	1.394E-05	-0.909
Organonitrogen compounds	1-Phenylethylamine	HMDB0002017	3.35	0.65	243.18	POS	C8H11N	7.736E-06	-0.665
Organic sulfonic acids and derivatives	Taurine	HMDB0000251	1.85	0.65	126.02	POS	C2H7NO3S	5.185E-03	0.486
Organic acids and derivatives	N-Methylethanolaminium phosphate	HMDB0060173	1.12	0.59	156.04	POS	C3H10NO4P	8.848E-03	0.396
Non-metal oxoanionic compounds	Pyrophosphate	HMDB0000250	2.72	0.64	176.94	NEG	H4O7P2	1.021E-03	-0.317
Methylmalonic acid	Methylmalonic acid	HMDB0000202	3.14	0.66	163.02	NEG	C4H6O4	9.672E-06	-0.876
Lactones	Dehydroascorbic acid	HMDB0001264	1.67	0.71	219.01	NEG	C6H6O6	1.743E-06	1.235
Keto acids and	Pyruvic acid	HMDB0000243	4.72	0.71	133.01	NEG	C3H4O3	1.722E-03	-0.404
Indoles and derivatives	5-Hydroxyindoleacetic acid	HMDB0000763	1.16	2.23	209.09	POS	C10H9NO3	8.649E-08	2.389

Cinnamic acids and derivatives	2-Hydroxycinnamic acid	HMDB0002641	3.31	0.75	182.08	POS	C9H8O3	2.071E-04	-0.437
Carboxylic acids and derivatives	Oxidized glutathione	HMDB0003337	3.45	0.71	611.14	NEG	C20H32N6O12S2	3.258E-02	-0.339
	Phosphoserine	HMDB0000272	1.05	0.65	186.02	POS	C3H8NO6P	3.362E-03	-0.406
	N2-Succinyl-L-ornithine	HMDB0001199	1.14	1.09	233.11	POS	C9H16N2O5	5.195E-03	-0.415
	Methionine sulfoxide	HMDB0002005	1.94	0.75	166.05	POS	C5H11NO3S	3.113E-02	-0.418
	Glutamylglutamic acid	HMDB0028818	3.21	0.71	277.10	POS	C10H16N2O7	1.958E-03	-0.440
	L-Aspartyl-4-phosphate	HMDB0012250	1.73	0.74	214.01	POS	C4H8NO7P	6.440E-03	-0.442
	N-Acetyl-L-aspartic acid	HMDB0000812	21.38	0.91	174.04	NEG	C6H9NO5	9.044E-05	-0.464
	N-Acetyl-L-alanine	HMDB0000766	2.68	0.91	130.05	NEG	C5H9NO3	7.934E-05	-0.477
	Maleic acid	HMDB0000176	1.60	1.07	115.00	NEG	C4H4O4	1.019E-02	-0.482
	3-Nitrotyrosine	HMDB0001904	1.04	0.71	244.09	POS	C9H10N2O5	1.322E-04	-0.487
	N2-gamma-Glutamylglutamine	HMDB0011738	1.05	0.67	274.10	NEG	C10H17N3O6	6.478E-04	-0.497
	L-Tyrosine	HMDB0000158	4.86	1.13	182.08	POS	C9H11NO3	2.698E-05	-0.515
	N-Acetylaspartylglutamic acid	HMDB0001067	9.88	1.12	303.08	NEG	C11H16N2O8	3.346E-04	-0.571
	4-Guanidinobutanoic acid	HMDB0003464	1.16	0.97	146.09	POS	C5H11N3O2	7.126E-06	-0.690
	gamma-Glutamylglutamic acid	HMDB0011737	2.82	0.91	277.10	POS	C10H16N2O7	2.219E-04	-0.713
	Garcinia acid	HMDB0031159	3.18	0.68	207.01	NEG	C6H8O8	4.653E-04	-0.759
	N-Acetyl-L-aspartic acid	HMDB0000812	2.16	0.91	349.09	NEG	C6H9NO5	9.172E-05	-0.812
	N-a-Acetyl-L-arginine	HMDB0004620	1.03	0.97	217.13	POS	C8H16N4O3	2.093E-04	-0.850
	Pipecolic acid	HMDB0000070	1.13	0.97	130.09	POS	C6H11NO2	2.895E-03	-0.859
	Succinic acid	HMDB0000254	2.79	1.15	117.02	NEG	C4H6O4	3.751E-03	-1.426
5'-deoxyribonucleosides	S-Adenosylhomocysteine	HMDB0000939	1.56	1.09	385.13	POS	C14H20N6O5S	7.648E-04	-0.382

12(R)-HETE, (5Z,8Z,10E,14Z)-(12R)-12-Hydroxyeicosa-5,8,10,14-tetraenoic acid; LPA, lysophosphatidic acid; LPC, lysophosphatidylcholine; LPE, lysophosphatidylethanolamine; LPG, lysophosphatidylglycerol; LPI, lysophosphatidylinositol; LyPS, lysophosphatidylserine; PA, phosphatidic acid; PC, phosphatidylcholine; PE, phosphatidylethanolamine; PG, phosphatidylglycerol; PS, phosphatidylserine; VIP, variable importance in projection; FC, Fold change; RT, Retention time; Compound ID was mainly exhibited based on the Human Metabolome Database (http://www.hmdb.ca), and LIPID MAPS (http://www.lipidmaps.org).

Table S5. Significantly differential metabolites responsible for the discrimination of two groups (LPS-F *vs.* CON-F)

Class	Name	Compound ID	VIP	RT (min)	m/z	Ion mode	Formula	*P*	Log2FC
Sphingolipids	Sphinganine	HMDB0000269	1.10	5.86	302.31	POS	C18H39NO2	3.022E-02	-0.411
	GlcCer(d18:1/24:1)	HMDB0004975	6.09	11.49	854.67	NEG	C48H91NO8	4.281E-02	-0.496
	Cer(d18:1/18:1)	HMDB0004948	8.75	10.40	608.53	NEG	C36H69NO3	7.408E-03	-0.745
	Cer(d18:1/18:0)	HMDB0004950	15.74	10.90	610.54	NEG	C36H71NO3	9.246E-03	-0.754
Ribonucleoside 3'-phosphates	Adenosine 3'-monophosphate	HMDB0003540	7.54	0.71	348.07	POS	C10H14N5O7P	4.710E-02	0.584
Purine nucleosides	Guanosine	HMDB0000133	1.73	1.28	282.08	NEG	C10H13N5O5	2.763E-02	-0.362
Organooxygen compounds	Pantothenic Acid	HMDB0000210	8.46	2.30	220.12	POS	C9H17NO5	1.054E-04	0.777
	5-Aminoimidazole ribonucleotide	HMDB0001235	3.94	0.63	296.07	POS	C8H14N3O7P	2.867E-02	0.458
	D-Sedoheptulose 7-phosphate	HMDB0001068	1.80	0.64	289.03	NEG	C7H15O10P	4.459E-02	0.330
	Galactose 1-phosphate	HMDB0000645	4.70	0.64	259.02	NEG	C6H13O9P	5.060E-02	0.303
	D-Glucose	HMDB0000122	1.94	0.65	203.05	POS	C6H12O6	1.387E-02	0.301
Organic phosphoric acids and derivatives	O-Phosphoethanolamine	HMDB0000224	1.03	0.65	164.01	POS	C2H8NO4P	3.080E-03	0.261
Indoles and derivatives	5-Hydroxyindoleacetic acid	HMDB0000763	1.50	2.23	209.09	POS	C10H9NO3	2.518E-04	1.831
Hydroxy acids and derivatives	Malic acid	HMDB0000156	1.04	0.71	152.06	POS	C4H6O5	3.779E-02	-0.334
Glycerophospholipids	PC(46:2)	HMDB0008585	1.62	0.57	924.74	NEG	C54H104NO8P	1.035E-03	1.726
	PS(39:4)	LMGP03010247	1.43	10.11	870.55	NEG	C45H80NO10P	6.796E-04	0.908
	PA(O-16:0/20:2)	LMGP10020014	1.64	8.53	704.56	POS	C39H75O7P	2.929E-02	0.769
	PS(39:3)	LMGP03010275	1.91	10.44	872.56	NEG	C45H82NO10P	1.775E-02	0.359
	PS(40:6)	HMDB0012444	10.25	3.63	836.54	POS	C46H78NO10P	3.954E-02	0.342
	PS(41:3)	LMGP03010508	1.79	10.91	900.60	NEG	C47H86NO10P	2.889E-02	0.265
	PS(37:2)	HMDB0116749	1.03	10.53	846.55	NEG	C43H80NO10P	4.495E-02	0.263
	PC(P-20:0/21:0)	LMGP01030099	2.11	0.59	888.70	NEG	C49H98NO7P	2.997E-02	0.261
	LPC(20:3)	HMDB0010393	2.04	5.53	546.36	POS	C28H52NO7P	3.076E-02	-0.264
	LPC(22:6)	HMDB0010404	2.80	6.65	612.33	NEG	C30H50NO7P	2.305E-02	-0.266

Glycerophospholipids	PE(O-18:0/19:1)	LMGP02020053	2.35	10.76	790.60	NEG	C42H84NO7P	4.174E-02	-0.283
	LPE(18:0)	HMDB0011130	2.11	7.86	480.31	NEG	C23H48NO7P	8.256E-03	-0.298
	LPC(18:1)	HMDB0002815	3.29	7.19	566.35	NEG	C26H52NO7P	4.379E-02	-0.311
	PE(18:1/P-18:1)	HMDB0009050	3.90	10.79	726.55	NEG	C41H78NO7P	4.761E-02	-0.312
	PE(20:4/P-18:0)	HMDB0009412	4.36	10.70	750.54	NEG	C43H78NO7P	2.510E-02	-0.333
	PE(22:6/P-18:0)	HMDB0009709	6.09	10.51	774.54	NEG	C45H78NO7P	1.485E-02	-0.371
	PE(44:10)	HMDB0009605	2.58	9.75	838.54	NEG	C49H78NO8P	5.370E-03	-0.380
	PE(20:4/P-16:0)	HMDB0009411	2.82	10.27	722.51	NEG	C41H74NO7P	2.554E-02	-0.385
	PE(40:7)	HMDB0009685	3.63	9.91	788.52	NEG	C45H76NO8P	5.996E-03	-0.400
	PE(22:4/P-16:0)	HMDB0009609	3.34	10.57	750.54	NEG	C43H78NO7P	6.452E-03	-0.412
	PE(36:2)	HMDB0009025	3.91	10.57	742.54	NEG	C41H78NO8P	9.690E-04	-0.425
	PE(38:6)	HMDB0008946	7.51	9.91	762.51	NEG	C43H74NO8P	1.410E-02	-0.435
	PE(40:4)	HMDB0009009	3.39	10.81	794.57	NEG	C45H82NO8P	1.833E-02	-0.437
	LPE(20:4)	HMDB0011517	3.36	6.67	500.28	NEG	C25H44NO7P	4.188E-03	-0.476
	PE(22:4/P-18:1)	HMDB0009611	3.10	10.56	776.56	NEG	C45H80NO7P	7.953E-03	-0.506
	PE(38:4)	HMDB0009322	2.40	11.07	812.54	NEG	C43H78NO8P	7.094E-03	-0.560
	PE(22:4/P-18:0)	HMDB0009610	4.37	11.03	778.58	NEG	C45H82NO7P	1.549E-03	-0.573
	PE(18:0/P-18:1)	HMDB0009017	5.22	11.32	728.56	NEG	C41H80NO7P	7.372E-03	-0.574
	PE(36:1)	HMDB0008992	5.29	11.07	744.56	NEG	C41H80NO8P	5.412E-03	-0.593
	LPA(20:1)	HMDB0062305	1.88	7.56	465.30	POS	C23H45O7P	4.426E-03	-0.837
	LPA(18:0)	HMDB0007854	1.00	7.43	439.28	POS	C21H43O7P	5.375E-03	-1.028
	Glycerophosphocholine	HMDB0000086	7.49	0.66	258.11	POS	C8H20NO6P	2.006E-02	0.416
Fatty Acyls	cis-5-Tetradecenoylcarnitine	HMDB0002014	1.94	4.99	370.30	POS	C21H39NO4	2.587E-02	0.834
	Dodecanoylcarnitine	HMDB0002250	1.62	4.61	344.28	POS	C19H37NO4	3.790E-02	0.663
	trans-Hexadec-2-enoyl carnitine	HMDB0006317	3.24	5.90	398.33	POS	C23H43NO4	3.670E-02	0.558
	12-hydroxy lauric acid	HMDB0002059	1.04	3.11	234.21	POS	C12H24O3	3.070E-03	-0.357
	Tetracosanoic acid	HMDB0002003	1.76	10.35	367.36	NEG	C24H48O2	2.120E-02	-0.414

Sterol Lipids	21-Deoxycortisol	HMDB0004030	3.21	3.51	347.22	POS	C21H30O4	1.333E-02	1.051
	5a-Tetrahydrocortisol	HMDB0000526	1.35	4.15	384.27	POS	C21H34O5	3.545E-02	0.808
	11-Dehydrocorticosterone	HMDB0004029	1.71	3.41	345.21	POS	C21H28O4	1.865E-02	1.360
	Progesterone	HMDB0001830	1.51	4.84	315.23	POS	C21H30O2	1.863E-02	-1.812
	Allopregnanolone	HMDB0001449	1.31	2.83	341.24	POS	C21H34O2	4.119E-02	0.929
	Pregnenolone	HMDB0000253	1.91	2.94	339.23	POS	C21H32O2	3.162E-02	-1.353
	Estradiol	HMDB0000151	1.09	2.47	295.17	POS	C18H24O2	3.696E-03	0.857
	Dehydroepiandrosterone	HMDB0000077	1.78	5.12	333.21	NEG	C19H28O2	1.784E-02	0.952
Carboxylic acids and derivatives	Ophthalmic acid	HMDB0005765	1.55	1.07	290.13	POS	C11H19N3O6	1.861E-03	1.307
	L-Glutamine	HMDB0000641	2.18	0.65	169.06	POS	C5H10N2O3	1.055E-03	0.311

LPA, lysophosphatidic acid; LPC, lysophosphatidylcholine; LPE, lysophosphatidylethanolamine; PA, phosphatidic acid; PC, phosphatidylcholine; PE, phosphatidylethanolamine; PS, phosphatidylserine; VIP, variable importance in projection; FC, Fold change; RT, Retention time; Compound ID was mainly exhibited based on the Human Metabolome Database (HMDB, http://www.hmdb.ca), and LIPID MAPS (http://www.lipidmaps.org).

Table S6. Significantly differential metabolites responsible for the discrimination of two groups (LPS-F *vs.* LPS-M).

Class	Name	Compound ID	VIP	RT (min)	m/z	Ion mode	Formula	P (T test)	Log2FC
Sterol Lipids	5α-Pregnane-3,20-dione	HMDB0003759	1.22	4.44	317.25	POS	C21H32O2	9.939E-04	4.669
	Tetrahydrocortisone	HMDB0000903	1.39	3.88	382.26	POS	C21H32O5	1.081E-05	4.406
	5α-Tetrahydrocortisol	HMDB0000526	1.02	4.15	384.27	POS	C21H34O5	1.443E-05	3.523
	Progesterone	HMDB0001830	1.24	4.84	315.23	POS	C21H30O2	5.480E-04	-0.982
	Allopregnanolone	HMDB0001449	1.28	2.83	341.24	POS	C21H34O2	1.268E-02	1.281
	12-Ketodeoxycholic acid	HMDB0000328	1.28	7.71	408.31	POS	C24H38O4	1.330E-02	1.763
Sphingolipids	Sphinganine	HMDB0000269	1.16	5.86	302.31	POS	C18H39NO2	9.401E-03	1.069
	GlcCer(d18:1/18:1)	HMDB0004970	3.02	11.82	726.59	POS	C42H79NO8	3.894E-02	1.047
	Sphingosine	HMDB0000252	3.05	5.53	300.29	POS	C18H37NO2	8.981E-03	0.838
Pteridines and derivatives	7,8-Dihydroneopterin	HMDB0002275	1.14	1.06	256.10	POS	C9H13N5O4	1.985E-02	-1.187
Organooxygen compounds	Pantothenic Acid	HMDB0000210	7.68	2.30	220.12	POS	C9H17NO5	9.317E-06	1.043
	5-Aminoimidazole ribonucleotide	HMDB0001235	1.84	0.63	296.07	POS	C8H14N3O7P	4.162E-02	0.417
Lactones	Dehydroascorbic acid	HMDB0001264	1.35	0.71	219.01	NEG	C6H6O6	4.645E-02	-0.437
Glycerophospholipids	LPI(18:0)	HMDB0240261	3.24	6.09	601.33	POS	C27H53O12P	1.692E-02	0.333
	PC(32:0)	LMGP01010537	5.58	10.50	778.56	NEG	C40H80NO8P	1.205E-02	0.323
	PS(39:2)	LMGP02010813	2.96	10.44	830.59	NEG	C44H84NO8P	1.827E-03	-0.307
	PS(39:3)	LMGP03010275	3.15	10.44	872.56	NEG	C45H82NO10P	2.599E-02	-0.541
	PS(P-29:0)	LMGP03030002	1.11	7.65	678.47	POS	C35H68NO9P	8.389E-03	-0.654
	PC(36:4)	HMDB0007983	7.97	11.66	804.55	POS	C44H80NO8P	8.877E-03	-0.682
Glycerolipids	TG(46:5)	LMGL03012657	1.54	10.61	813.62	NEG	C49H84O6	2.368E-02	0.407
	MG(17:0)	HMDB0072856	1.94	5.11	362.33	POS	C20H40O4	4.297E-03	-0.546
Fatty Acyls	Behenic acid	HMDB0000944	2.01	8.16	358.37	POS	C22H44O2	3.663E-02	1.045
	9-tridecynoic acid	LMFA01030602	1.49	7.85	228.20	POS	C13H22O2	2.195E-02	0.959
	24-hydroxy-tetracosanoic acid	LMFA01050213	1.37	8.18	402.39	POS	C24H48O3	4.876E-02	0.710
	16-Methylheptadecanoic acid	HMDB0031066	4.69	8.87	283.26	NEG	C18H36O2	2.844E-02	0.388
	Palmitic acid	HMDB0000220	1.72	8.29	255.23	NEG	C16H32O2	4.939E-02	0.341

Fatty Acyls	Stearoylethanolamide	HMDB0013078	1.44	7.87	328.32	POS	$C_{20}H_{41}NO_2$	4.720E-02	0.335
	16-Hydroxyhexadecanoic acid	HMDB0006294	2.49	4.27	290.27	POS	$C_{16}H_{32}O_3$	2.150E-02	-0.418
	20-hydroxy-eicosanoic acid	LMFA01050075	1.33	6.35	346.33	POS	$C_{20}H_{40}O_3$	6.254E-03	-0.439
	13,14-dihydroxy-docosanoic acid	LMFA01050211	1.28	6.29	390.36	POS	$C_{22}H_{44}O_4$	7.207E-03	-0.474
	12-Hydroxydodecanoic acid	HMDB0002059	1.21	3.11	234.21	POS	$C_{12}H_{24}O_3$	8.069E-03	-0.552
	ω-hydroxy myristic acid	LMFA01050044	1.26	3.64	262.24	POS	$C_{14}H_{28}O_3$	7.769E-03	-0.590
	2-Methylcitric acid	HMDB0000379	1.17	0.85	224.08	POS	$C_7H_{10}O_7$	2.537E-02	-0.658
Carboxylic acids and derivatives	Succinic acid	HMDB0000254	3.80	1.15	117.02	NEG	$C_4H_6O_4$	1.829E-02	1.096
	Ophthalmic acid	HMDB0005765	1.05	1.07	290.13	POS	$C_{11}H_{19}N_3O_6$	6.363E-03	1.034
	L-Methionine	HMDB0000696	2.97	0.75	150.06	POS	$C_5H_{11}NO_2S$	1.581E-02	0.396
Benzenoids	Vanylglycol	HMDB0001490	1.04	2.30	202.11	POS	$C_9H_{12}O_4$	2.596E-06	1.016

LPI, lysophosphatidylinositol; PC, phosphatidylcholine; PS, phosphatidylserine; TG, Triacylglycerols; MG, Monoradylglycerols; VIP, variable importance in projection; FC, Fold change; RT, Retention time; Compound ID was mainly exhibited based on the Human Metabolome Database (HMDB, http://www.hmdb.ca), and LIPID MAPS (http://www.lipidmaps.org).
