# Supplementary material for: The hypothalamic steroidogenic pathway mediates susceptibility to inflammation-evoked depression in female mice
Source: J Neuroinflammation. 2023 Dec 7;20:293. doi: 10.1186/s12974-023-02976-7 (PMC10704691; doi:10.1186/s12974-023-02976-7)

Fig. 5E Original western blot images

Original blot images of StAR protein

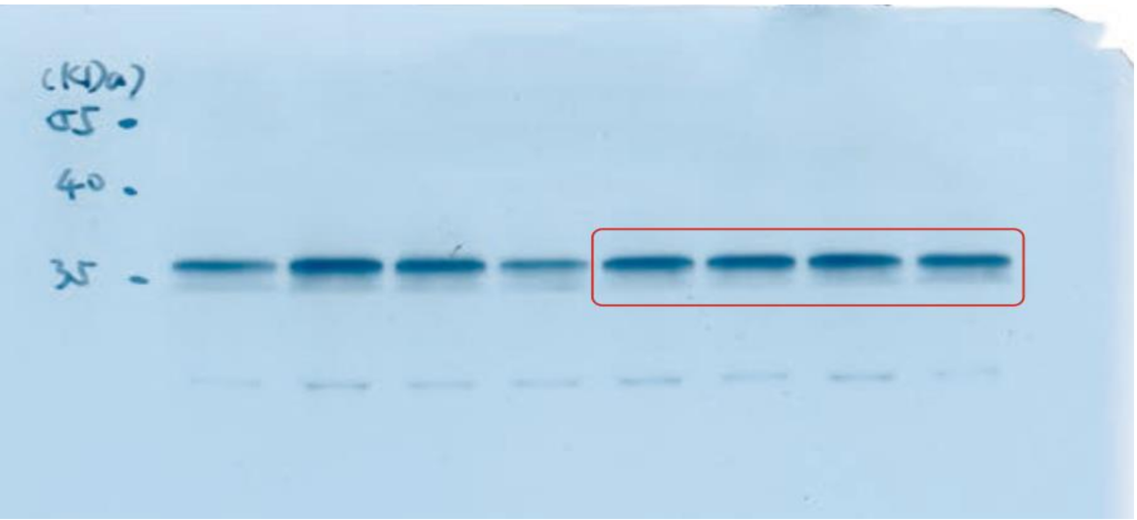

Original blot images of CYP11A1 protein

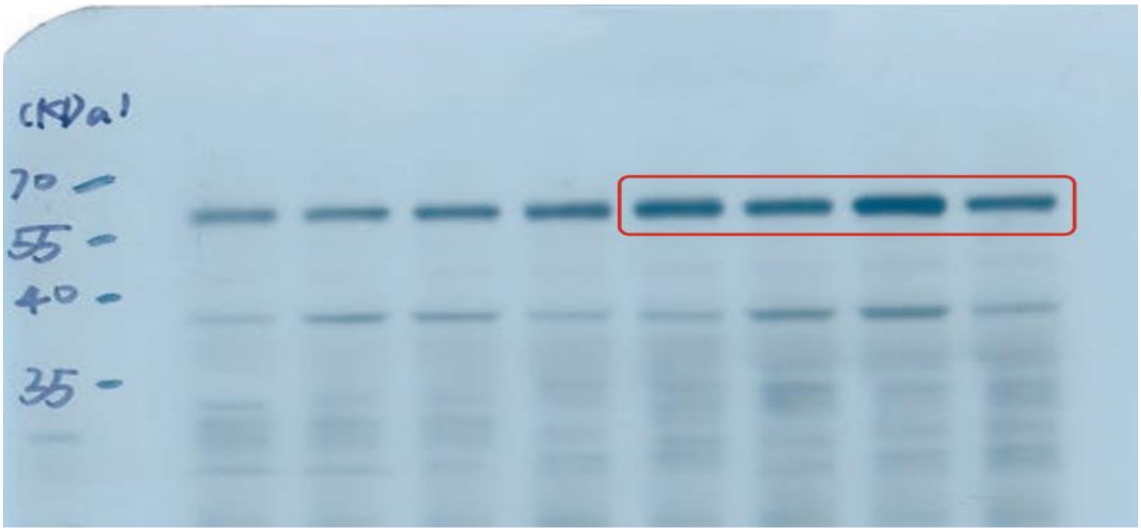

Original blot images of CYP11B1 protein

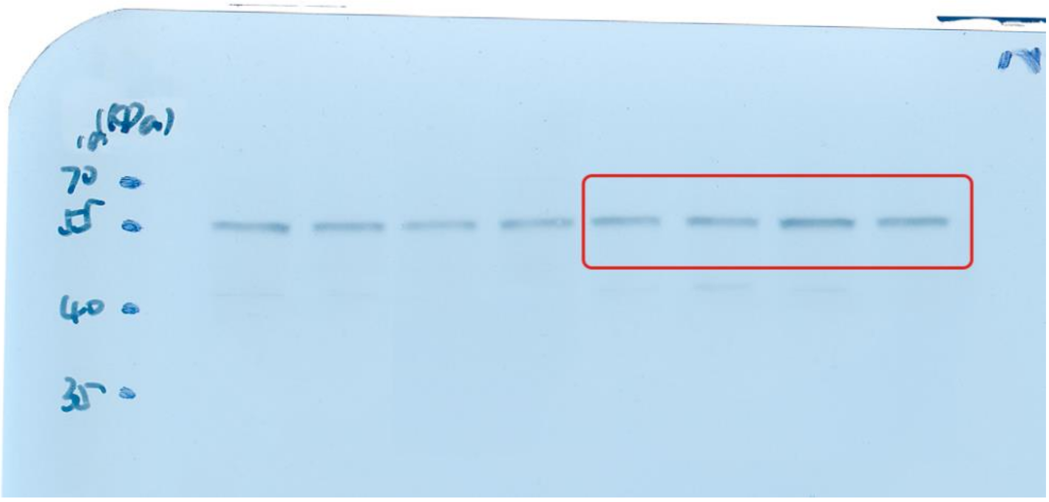

Original blot images of CYP11B2 protein

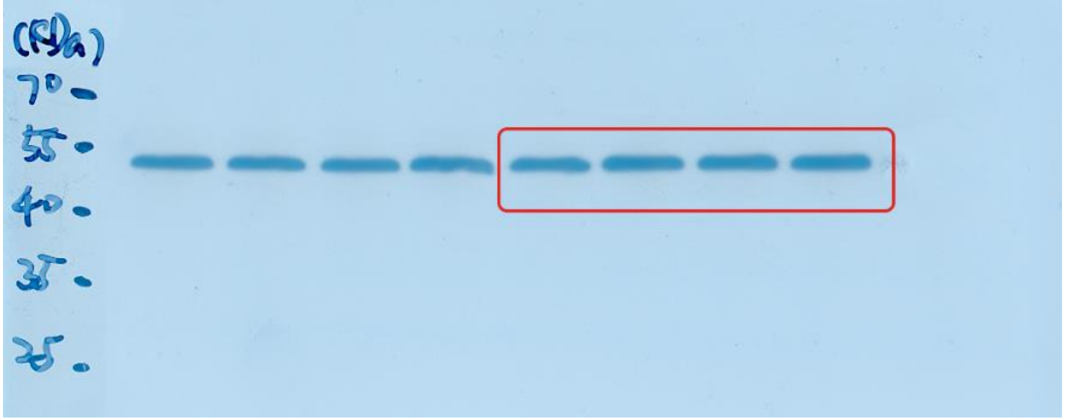

Original blot images of SRD5A1 protein

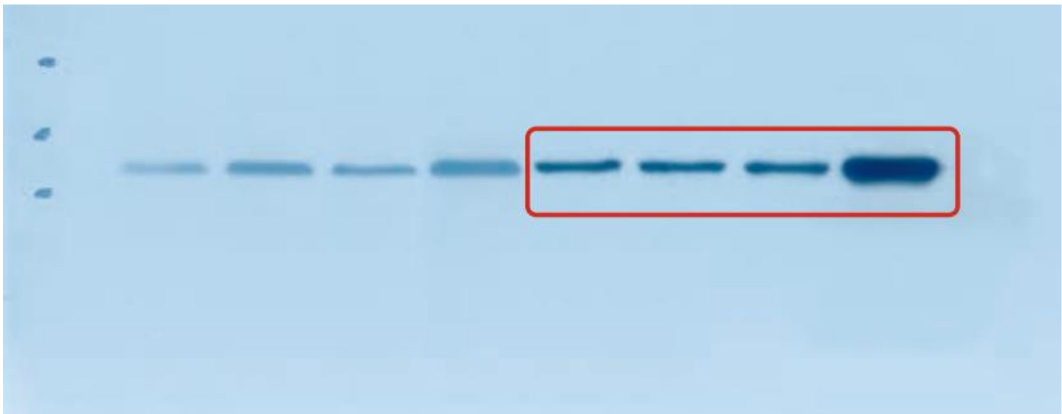

Original blot images of SRD5A2 protein

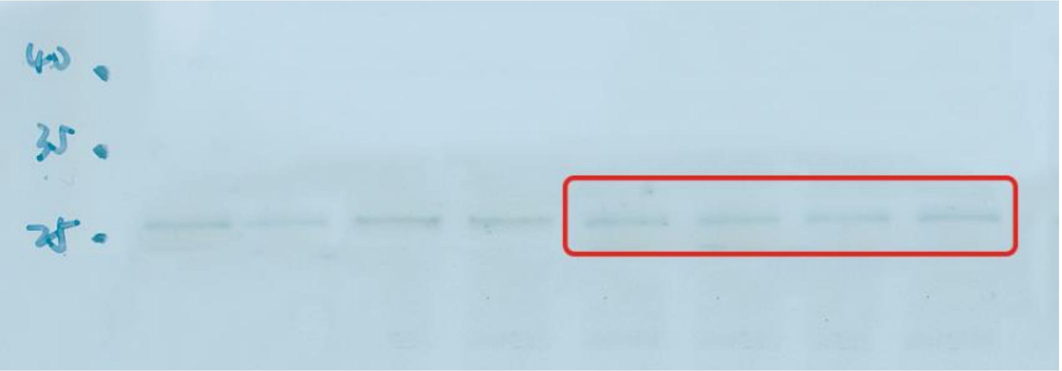

Original blot images of GAPDH protein

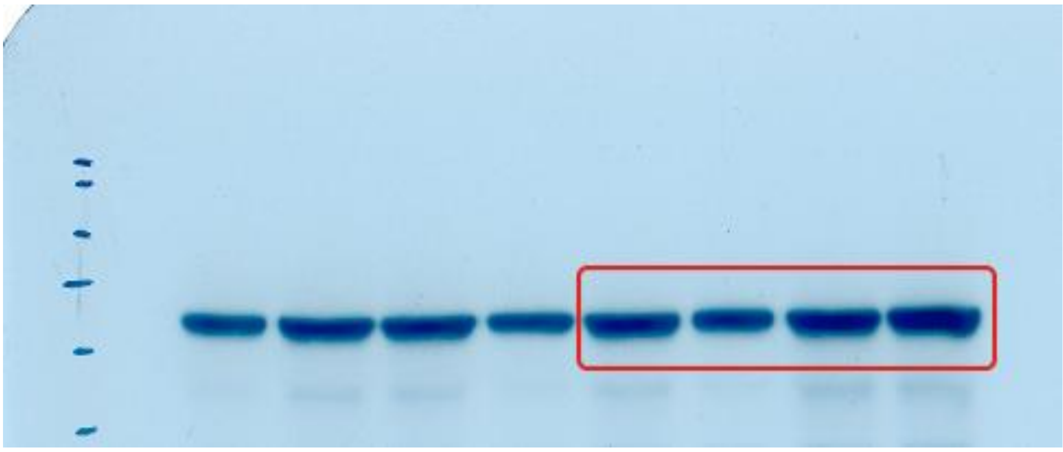

Supplement: Supplementary file 5 — Additional file 5. Original western blot image [file 12974_2023_2976_MOESM5_ESM.pdf]
